# The Spatiotemporal Heterogeneity of Tumor-Associated Stromal Cells: Reprogramming Plasticity to Unlock Precision Cancer Immunotherapy

**DOI:** 10.34133/cancomm.0002

**Published:** 2026-01-22

**Authors:** Yingying Lv, Tingfei Duan, Jinling Song, Shutong Liu, Zhaokai Zhou, Yuhao Ba, Siyuan Weng, Anning Zuo, Hui Xu, Peng Luo, Quan Cheng, Chuhan Zhang, Jingyuan Ning, Yukang Chen, Yuyuan Zhang, Zaoqu Liu, Xinwei Han

**Affiliations:** ^1^ Department of Interventional Radiology, The First Affiliated Hospital of Zhengzhou University, Zhengzhou, Henan, P. R. China.; ^2^ Department of Pediatrics, The First Affiliated Hospital of Zhengzhou University, Zhengzhou, Henan, P. R. China.; ^3^ Department of Pediatrics, The Third Affiliated Hospital of Zhengzhou University, Zhengzhou, Henan, P. R. China.; ^4^ Division of Pulmonology, Department of Pediatrics, The Third Affiliated Hospital of Zhengzhou University, Zhengzhou, Henan, P. R. China.; ^5^ Department of Urology, The Second Xiangya Hospital of Central South University, Changsha, Hunan, P. R. China.; ^6^ Department of Urology, The First Affiliated Hospital of Zhengzhou University, Zhengzhou, Henan, P. R. China.; ^7^ Department of Oncology, Zhujiang Hospital, Southern Medical University, Guangzhou, Guangdong, P. R. China.; ^8^ Department of Neurosurgery, Xiangya Hospital, Central South University, Changsha, Hunan, P. R. China.; ^9^ Department of Oncology, The First Affiliated Hospital of Zhengzhou University, Zhengzhou, Henan, P. R. China.; ^10^ Key Laboratory of Immune Mechanism and Intervention on Serious Disease in Hebei Province, Department of Immunology, Hebei Medical University, Shijiazhuang, P. R. China.; ^11^ Interventional Institute of Zhengzhou University, Zhengzhou, Henan, P. R. China.; ^12^ Interventional Treatment and Clinical Research Center of Henan Province, Zhengzhou, Henan, P. R. China.; ^13^ Institute of Basic Medical Sciences, Chinese Academy of Medical Sciences and Peking Union Medical College, Beijing, P. R. China.

## Abstract

Tumor-associated stromal cells (TASCs) are key architects of the tumor microenvironment (TME), playing a vital role in tumor development, metastasis, and therapeutic response. Their spatiotemporal heterogeneity, characterized by dynamic phenotypic plasticity, diverse cellular subtypes, and distinct spatial distributions, offers profound insights into tumor behavior and paves the way for innovative therapy development. In particular, stromal–immune interactions reveal the powerful capacity of TASCs to shape the immune landscape, highlighting their potential as targets in immunotherapy. Despite growing evidence in functional diversity, precise mechanisms underlying the temporal evolution and spatial organization of TASCs remain elusive, impeding clinical translation. This review delved into the molecular signatures and functional states of TASCs, emphasizing their roles in tumor dynamics and therapeutic resistance. We also discussed innovative strategies targeting the plasticity of TASCs to reverse immune evasion and potentiate immune-mediated tumor eradication. Future studies should prioritize identifying spatially resolved and mechanically defined biomarkers with multi-omics and machine learning approaches, enabling a comprehensive understanding of TASCs to bridge the gap from bench to bedside.

## Introduction

**Fig. 1.**
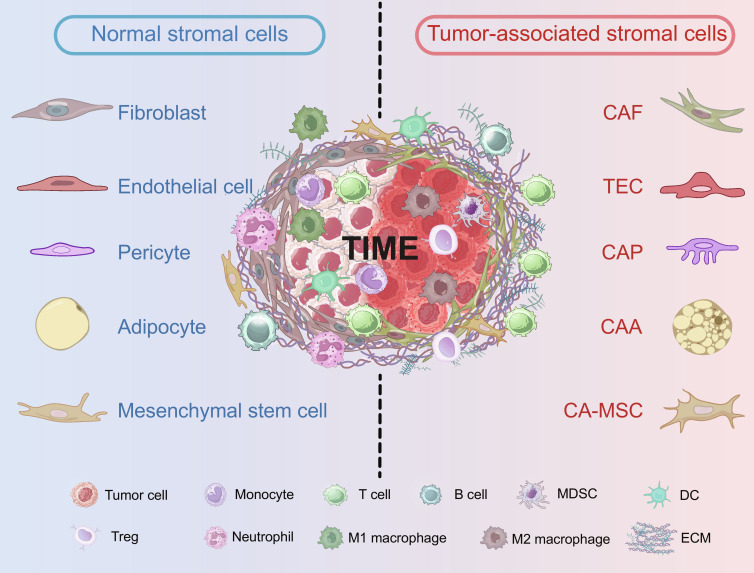
Normal stromal cells versus TASCs. TASCs, including CAFs, TECs, CAPs, CAAs, and CA-MSCs, are dynamically reprogrammed by neoplastic cells. These cells exhibited distinct morphological, functional, and cellular interactions compared to their normal counterparts. In normal tissue, stromal cells exert antitumor effects and communicate with protective immune cells (e.g., monocytes, neutrophils, and M1 macrophages) to suppress tumor growth. In contrast, TASCs actively foster tumor progression. They orchestrate the TIME by inducing M2 macrophage polarization, recruiting Tregs, and so on. CAAs, cancer-associated adipocytes; CAFs, cancer-associated fibroblasts; CA-MSCs, carcinoma-associated mesenchymal stem cells; CAPs, cancer-associated pericytes; DCs, dendritic cells; ECM, extracellular matrix; MDSCs, myeloid-derived suppressor cells; TASCs, tumor-associated stromal cells; TECs, tumor-associated endothelial cells; TIME, tumor immune microenvironment; Tregs, regulatory T cells.

The tumor microenvironment (TME) has emerged as an indispensable component of the complex tumor ecosystem, spanning tumor initiation, development, and metastasis [1]. Tumor-associated stromal cells (TASCs) are prominent cells of the TME and include cancer-associated fibroblasts (CAFs), tumor-associated endothelial cells (TECs), cancer-associated pericytes (CAPs), cancer-associated adipocytes (CAAs), and carcinoma-associated mesenchymal stem cells (CA-MSCs; Fig. 1). These stromal components display a wide range of heterogeneity regarding their origins, subtypes, biomarkers, and functions (Table 1). Stromal cells modulate tumor progression through multiple mechanisms, including providing structural support, orchestrating immunosuppressive environments, and influencing tumor cell behaviors through the secretion of growth factors, cytokines, enzymes, and extracellular matrices (ECMs) [2]. In addition, other nonimmune stromal cells, including Schwann cells, astrocytes, and osteoblasts, are present in specialized TMEs [3,4]. These cells are not described in this review, as they are relatively understudied and excluded from mainstream discussions.

**Table 1.** The heterogeneity of tumor-associated stromal cells

Cell type	Overview	Origins	Markers	Refs.
CAAs	Spindle-shaped adipocytes at the tumor invasion front are involved in metabolic reprogramming	Normal adipocytes, mesenchymal stem cells	Down-regulation of leptin, adiponectin, resistin, FABP4, and up-regulation of IL-6, TNF-α	[239–241]
CAFs	CAFs are major stromal components in solid tumors (e.g., breast, pancreatic, and liver cancers), contributing to poor prognosis	Tissue-resident fibroblasts (e.g., CD26^+^ fibroblasts), nonfibroblast lineages (e.g., endothelial cells, pericytes, adipocytes), bone marrow-derived cells (e.g., macrophages)	Vimentin, α-SMA, FSP1, FAP, PDGFRα/β	[242–244]
CAPs	CAPs are located on capillaries/venules and interact with TECs to maintain vascular stability	Tissue-specific origins (mesoderm, neural crest), EMT-transformed cancer cells, mesenchymal/myeloid precursors	Desmin, NG2, PDGFRβ, RGS5, CD13, CD146	[2,245–247]
CA-MSCs	CA-MSCs are multipotent adult stem cells with clinical potential in cell therapy	Bone marrow, umbilical cord (Wharton’s jelly), adipose tissue, heart, spleen	Common marker (CD105, CD73, CD90), osteogenic marker (RUNX2), adipogenic marker (PPAR-γ), chondrogenic marker (SOX9)	[248–253]
TECs	TECs regulate tumor vascularization and tertiary lymphoid structure formation	Vascular/endothelial progenitor cells, tumor stem cells, and tumor cells	PLVAP, CCL2, ICAM1, PECAM, CLDN5, VEGFR2	[16,129,254]

α-SMA, α-smooth muscle actin; BM-MSC, bone marrow-derived mesenchymal stem cells; CA-MSCs, carcinoma-associated mesenchymal stem cells; CAFs, cancer-associated fibroblasts; CAAs, cancer-associated adipocytes; CAPs, cancer-associated pericytes; CCL2, C–C motif chemokine ligand 2; CD, cluster of differentiation; CLDN5, claudin-5; EC, endothelial cell; EMT, epithelial–mesenchymal transition; FABP4, fatty acid-binding protein 4; FAP, fibroblast activation protein; FSP1, fibroblast specific protein 1; HEV, high endothelial venules; ICAM1, intercellular adhesion molecule 1; IL-6, interleukin-6; IMEC, immunoregulatory endothelial cells; NG2, neuron-glial antigen 2; PDGFRα/β, platelet-derived growth factor receptor α/β; PECAM, platelet endothelial cell adhesion molecule; PLVAP, plasmalemma vesicle associated protein; PPAR-γ, peroxisome proliferator-activated receptor γ; RGS5, regulator of G protein signaling 5; RUNX2, runt-related transcription factor 2; SOX9, SRY-box transcription factor 9; TASCs, tumor-associated stromal cells; TECs, tumor-associated endothelial cells; TNF-α, tumor necrosis factor-α; VEGFR2, vascular endothelial growth factor receptor 2; WJ-MSC, Wharton’s jelly-derived mesenchymal stem cells

**Fig. 2.**
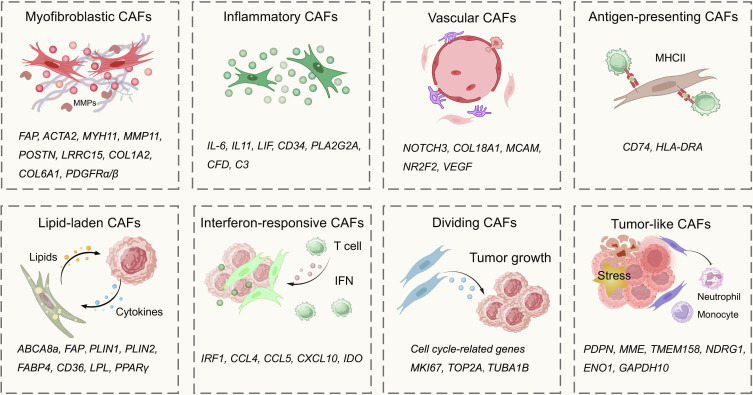
The main subtypes of CAFs. Advances in single-cell omics have led to the identification of increasingly diverse CAF subtypes across various tumor types. This figure illustrates 8 common CAF subtypes, along with their key functions and marker genes. Myofibroblastic CAFs are pivotal in ECM remodeling; inflammatory CAFs secrete pro-inflammatory factors, inhibiting tumor immune responses; antigen-presenting CAFs express MHC class II, enhancing tumor immunity; lipid-laden CAFs provide metabolic support; interferon-responsive CAFs are sensitive to interferon and engage in immunomodulatory crosstalk with T cells; dividing CAFs, characterized by cell cycle-related genes, support tumor growth; tumor-like CAFs acquire immunosuppressive properties and secrete IL-6 and IL-8 to suppress neutrophils and monocytes. This functional heterogeneity allows CAFs to orchestrate nearly every aspect of tumor progression, making them a compelling therapeutic target. ABCA8a, ATP-binding cassette subfamily A member 8; ACTA2, actin α2; CAFs, cancer-associated fibroblasts; CCL4, C–C motif chemokine ligand 4; CCL5, C–C motif chemokine ligand 5; C3, complement component 3; CFD, complement factor D; CD34, cluster of differentiation 34; CD36, cluster of differentiation 36; CD74, cluster of differentiation 74; COL1A2, collagen type I α2 chain; COL18A1, collagen type XVIII α1 chain; COL6A1, collagen type VI α1 chain; CXCL10, C–X–C motif chemokine ligand 10; ECM, extracellular matrix; ENO1, enolase 1; FABP4, fatty acid binding protein 4; FAP, fibroblast activation protein; GAPDH10, glyceraldehyde-3-phosphate dehydrogenase 10; HLA-DRA, major histocompatibility complex, class II, DRα; IFN, interferon; IDO, indoleamine 2,3-dioxygenase; IL-6, interleukin-6; IL-8, interleukin-8; IL-11, interleukin-11; IRF1, interferon regulatory factor 1; LIF, leukemia inhibitory factor; LRRC15, leucine rich repeat containing 15; LPL, lipoprotein lipase; MCAM, melanoma cell adhesion molecule; MME, membrane metalloendopeptidase; MKI67, marker of proliferation Ki-67; MMP11, matrix metallopeptidase 11; MYH11, myosin heavy chain 11; NDRG1, N-myc downstream regulated 1; NOTCH3, notch receptor 3; NR2F2, nuclear receptor subfamily 2 group F member 2; PDPN, podoplanin; PDGFRα/β, platelet-derived growth factor receptor α/β; PLA2G2A, phospholipase A2 group IIA; PLIN1, perilipin 1; PLIN2, perilipin 2; POSTN, periostin; PPARγ, peroxisome proliferator activated receptor γ; TUBA1B, tubulin α1b; TMEM158, transmembrane protein 158; TOP2A, DNA topoisomerase IIα; VEGF, vascular endothelial growth factor.

Single-cell analysis and spatial histology techniques have revealed the diversity of TASCs. The subtype heterogeneity of CAFs and TECs has been extensively explored, but the heterogeneity of other cells remains unclear. CAFs, as the mainstay of TASCs, can be divided into the following key types: myofibroblastic CAFs (myCAFs), inflammatory CAFs (iCAFs), vascular CAFs (vCAFs), antigen-presenting CAFs (apCAFs), and lipid-laden CAFs (lpCAFs). The heterogeneity of CAFs was initially identified in pancreatic ductal adenocarcinoma (PDAC), in which myCAFs highly expressed α-smooth muscle actin (α-SMA) and fibroblast activation protein-α (FAP). myCAFs promote fibrosis and remodel the ECM [5]. iCAFs are characterized by high expression of inflammatory factors and low expression of myofibroblast markers, thus contributing to the immunosuppressive microenvironment [6]. apCAFs, which express major histocompatibility complex (MHC) class II molecules but not classical costimulatory molecules, were first identified in murine models of PDAC [7]. This unique phenotype was later confirmed in human breast and lung cancer [8,9]. vCAFs, first systematically identified as a separate subpopulation of cells in the mouse mammary carcinoma (MMTV-PyMT) model, exhibit vascular features and angiogenesis-related gene expression profiles [10]. lpCAFs are characterized by lipid storage and transport, which accelerate malignant tumor progression through metabolic interactions [11,12]. Based on single-cell RNA sequencing (scRNA-seq) analyses of cross-cancer datasets, an increasing number of subgroups have emerged, further resolving the complexity and heterogeneity of CAFs [13,14]. Eight CAF subsets, which are widely explored across diverse tumor contexts, are shown in Fig. 2. Analogously, the taxonomy of annotated TEC subpopulations has been refined, mainly encompassing arterial TECs, capillary TECs, venous TECs, tip cells [15], activated postcapillary veins (PCVs), lymphatic TECs [16], and immunomodulatory TECs [17].

Novel computational frameworks leveraging single-cell omics data now enable predictions of intractable biological processes, such as cell-to-cell interaction networks and spatiotemporal gene expression dynamics. CellChat, as a competent tool for cellular interactions, is important for exploring the interaction networks among TASCs, tumor cells, and immune cells [18]. Trajectory analysis, RNA velocity, and dynamic scRNA-seq capture the dynamic changes in TASCs and increase the understanding of complex spatiotemporal heterogeneity [19,20]. From a temporal perspective, as tumors progress, the functions of stromal cells evolve as follows: (a) orchestrating tumor angiogenesis, providing oxygen and nutrition to support tumor growth [2], (b) metabolic reprogramming to supply energy and shape the TME [21], (c) participating in epithelial–mesenchymal transition (EMT) and endowing tumor cells with invasive properties [22], and (d) constructing the ECM and interacting with immune cells, leading to the formation of immunologically inert tumors [3]. Spatially, stromal cells in different tumor regions display distinct characteristics. In the hypoxic tumor core, iCAFs predominantly promote tumor growth, whereas at the boundary, myCAFs shift toward immunosuppression and facilitate tumor cell dissemination [4,23]. This spatiotemporal heterogeneity profoundly affects the functions of TASCs and, in turn, shapes the immune landscape and therapeutic responses.

To elucidate the spatiotemporal plasticity of TASCs, recent studies have increasingly emphasized the spatial architecture of tumors, aiming to map the spatial distribution, neighborhood topologies, and colocalization patterns of stromal constituents [24]. Spatial transcriptomics (ST) and its derivative platforms (e.g., Visium, MERSCOPE, CosMx, and Stereo-seq) provide insights into the cellular ecological niche of the TME while preserving spatial topological information [25]. A meta-analysis integrating 7 spatial-omics platforms across 10 cancer types has resolved the spatial subtypes of CAFs (S1 to S4), which exhibited differentiated spatial localization and diverse cellular interaction networks. S1-CAFs were adjacent to tumor cells, orchestrating collagen synthesis and matrix remodeling; S2-CAFs were localized in the peritumoral stroma and displayed a gene expression profile reminiscent of that of iCAFs; S3-CAFs encircled the vasculature and engaged in crosstalk with macrophages and neutrophils; and S4-CAFs were enriched in lymphoid aggregates and facilitated the recruitment of adaptive immune cells [26]. Despite these advances, most existing reviews focus on the molecular typing or functional heterogeneity of TASCs [27–29]. There is still a gap in the systematic exploration of their spatial heterogeneity. In this paper, we break through the traditional framework and systematically compare the spatial heterogeneity of TASCs from 3 perspectives: regional heterogeneity within the tumor, spatial evolution between the primary foci and metastases, and differences across various cancer types. Overall, this review provides a comprehensive overview of TASCs, focuses on their spatiotemporal heterogeneity, elucidates their dynamic associations with immune responses, and summarizes current therapeutic strategies. By dissecting these complex cellular interactions, we aim to establish a framework for novel targeted therapies and offer avenues to improve patient outcomes in clinical settings.

## Temporal Trajectory: Dynamic Evolution of TASCs

As dynamic entities, TASCs undergo marked functional transformations throughout the continuum of tumor development. Distinct subpopulations exert predominant influences at different stages.

### Tumor initiation phase: The activation of TASCs

**Fig. 3.**
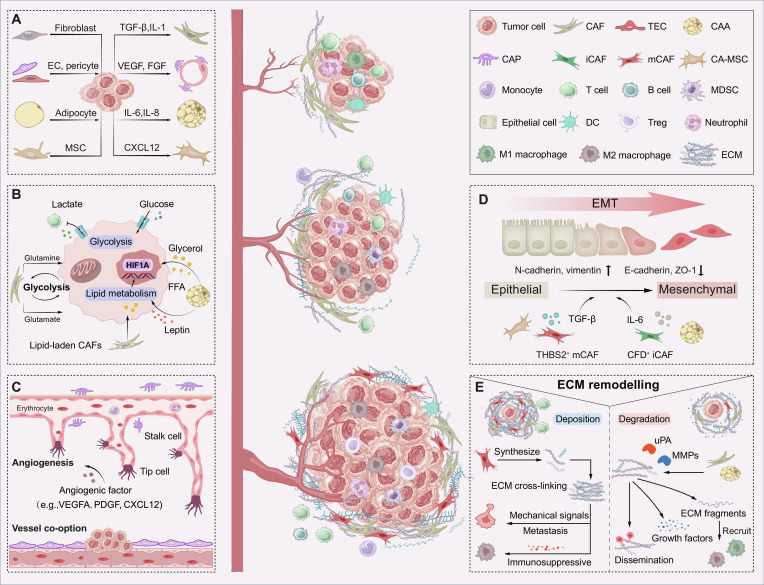
Temporal heterogeneity of TASCs in tumor progression. TASCs dynamically regulate tumor growth across distinct pathological stages through stage-specific mechanisms: (A) Tumor cells recruit and activate TASCs, which subsequently exert protumorigenic effects. Normal cells (left) can be reprogrammed into protumor states (right) by specific factors secreted from tumor cells. For instance, tumor-derived TGF-β and IL-1 activate CAFs; pro-angiogenic factors drive the formation of tumor vasculature by TECs and CAPs; IL-6 and IL-8 stimulate CAAs; CXCL12 accelerates the conversion of CA-MSCs. (B) TASCs undergo intrinsic metabolic reprogramming to modulate tumor metabolism and provide nutritional and energetic support. CAFs interplay with metabolic crosstalk by glycolysis and glutamate/glutamine metabolism. CAAs release glycerol, free fatty acids, and leptin, supporting the lipid metabolism in tumor cells. Lipid-laden CAFs also interplay with lipid metabolism. (C) TASCs facilitate angiogenesis through pro-angiogenic factors, including VEGFA, PDGF, and CXCL12. As a complementary pathway, tumor cells gain sufficient supplements by vessel co-option. (D) EMT is a critical process that endows tumor cells with invasive and metastatic capabilities. This is characterized by the up-regulation of mesenchymal markers (e.g., N-cadherin and vimentin) and the down-regulation of epithelial markers (e.g., E-cadherin and ZO-1). This transition is driven by various factors secreted by TASCs, such as TGF-β from CA-MSCs and THBS2^+^ mCAFs, and IL-6 from CAAs and CFD^+^ iCAFs. (E) TASCs participate in ECM remodeling to establish metastatic pathways. On the one hand, TASCs synthesize the main component of the ECM and trigger the maturation and stiffness. ECM promotes tumor progression through mechanical signals and immunosuppression. On the other hand, TASCs secrete uPA and MMPs, accelerating ECM degradation. The deconstructed ECM promotes tumor metastasis through the following mechanisms: relieving physical constraints, releasing cytokines, and recruiting immune cells via degraded fragments. apCAFs, antigen-presenting cancer-associated fibroblasts; CAAs, cancer-associated adipocytes; CAFs, cancer-associated fibroblasts; CA-MSCs, carcinoma-associated mesenchymal stem cells; CAPs, cancer-associated pericytes; CFD, complement factor D; CXCL12, C–X–C motif chemokine ligand 12; DCs, dendritic cells; EC, endothelial cell; ECM, extracellular matrix; EMT, epithelial–mesenchymal transition; FFA, free fatty acids; FGF, fibroblast growth factor; HIF1A, hypoxia inducible factor 1 subunit α; iCAFs, inflammatory cancer-associated fibroblasts; IL-1, interleukin-1; IL-6, interleukin-6; IL-8, interleukin-8; MDSCs, myeloid-derived suppressor cells; MMPs, matrix metalloproteinases; MSC, mesenchymal stem cell; myCAFs, myofibroblastic cancer-associated fibroblasts; PDGF, platelet-derived growth factor; TASCs, tumor-associated stromal cells; TECs, tumor-associated endothelial cells; TGF-β, transforming growth factor-β; THBS2, thrombospondin 2; Treg, regulatory T cells; uPA, urokinase-type plasminogen activator; vCAFs, vascular cancer-associated fibroblasts; VEGF, vascular endothelial growth factor; VEGFA, vascular endothelial growth factor A.

In the TME, cancer cells recruit and educate stromal cells, triggering a transition from indolent to active states. Different cytokines produced by tumor cells shape the heterogeneity of TASCs (Fig. 3A). In particular, the transformation of the CAF phenotypes relies on unique modes of action (Table 2). For CAPs and TECs that acquire invasive phenotypes, pro-angiogenic factors, such as vascular endothelial growth factor (VEGF) and fibroblast growth factor (FGF), and anti-angiogenic factors (e.g., endostatin and angiostatin) released by cancer cells are dominant regulators of angiogenic activity [30–33]. CAA activation is orchestrated by inflammatory factors, including interleukin-6 (IL-6) and interleukin-8 (IL-8) [34–36]. Mesenchymal stem cell (MSC) migration toward the tumor site is guided by a concentration gradient of factors. For example, cervical cancer cells recruited MSCs via the C–X–C motif chemokine ligand 12 (CXCL12)/C–X–C chemokine receptor type 4 (CXCR4) axis [37]. Upon arriving at the tumor site, MSCs further differentiate into CA-MSCs in response to inflammatory or hypoxic signals. A key example is that in breast cancer (BC), hypoxia-inducible factor (HIF) can educate MSCs to promote tumor progression [38].

**Table 2.** The heterogeneity of CAF subtypes across multiple dimensions

CAF stages	Subtype	Key determinants of functional heterogeneity	Refs.
Activation	apCAFs	IL-1, TGF-β	[255]
	iCAFs	IL-1, IFN-γ, HIF-1α	[100]
	myCAFs	TGF-β, PDGF, CREB3L1	[75,256]
	vCAFs	VEGF, FGF, angiopoietins	[257]
Angiogenesis	iCAFs	IL-8, CXCL1	[258]
	myCAFs	VEGF, FGF	
	vCAFs	VEGF, angiopoietin-1, and other angiogenic factors	
ECM remodeling	myCAFs (PDAC-specific)	COL10A1, COL11A1	[259]
	iCAFs (PDAC-specific)	COL14A1, COL3A1, COL6A1	
	Stellate-like CAFs (PDAC-specific)	COL4A1, COL4A2, COL5A3, COL14A1, COL18A1	
	apCAFs (PDAC-specific)	Less collagen expression, related to COL4A1	
Metabolism	iCAFs	Glycolysis and hypoxia signature	[260]
	myCAFs	OXPHOS and the TCA cycle	
	FAP^high^ CAFs	Glycolysis, arachidonic, and linoleic acid metabolism	[258]
	MCAM^high^ CAFs	OXPHOS and the TCA cycle	

PDAC-specific, the heterogeneity of collagen-secreting CAFs was investigated in PDAC, but its generalizability to other cancer types remains unclear; apCAFs, antigen-presenting cancer-associated fibroblasts; CAFs, cancer-associated fibroblasts; COL, collagen type; CREB3L1, cAMP responsive element binding protein 3 like 1; CXCL1, C–X–C motif chemokine ligand 1; ECM, extracellular matrix; FAP, fibroblast activation protein; FGF, fibroblast growth factor; HIF-1α, hypoxia inducible factor 1α; IFN-γ, interferon-γ; IL, interleukin; iCAFs, inflammatory cancer-associated fibroblasts; MCAM, melanoma cell adhesion molecule; myCAFs, myofibroblastic cancer-associated fibroblasts; OXPHOS, oxidative phosphorylation; PDAC, pancreatic ductal adenocarcinoma; PDGF, platelet-derived growth factor; TCA cycle, tricarboxylic acid cycle; TGF-β, transforming growth factor-β; VEGF, vascular endothelial growth factor; vCAFs, vascular cancer-associated fibroblasts

Notably, epigenetic alterations in tumor cells also reprogram TASCs. In pancreatic cancer, the ectopic enrichment of histone H3 lysine 27 (H3K27) acetylation in tumor cells has been observed during the transition of lpCAFs. This epigenetic mechanism triggered bone morphogenetic protein 2 (BMP2) signaling, reinforcing the protumorigenic phenotype of CAFs [39]. In addition, noncoding RNAs also engage in the regulation of stromal cell behaviors. miR-204-5p from tumor cells up-regulated hypoxia-inducible factor 1A (*HIF1A*) expression in white adipose tissue, leading to lipolysis and CAA maturation in engineered mice with BC [40].

### Tumor development phase: Dynamic progression of TASCs

#### TASC metabolic reprogramming regulates tumor development

As nurse cells, TASCs provide nutrients and energy for highly proliferative cancer cells. The Warburg effect, a crucial hallmark of cancer, indicates that cancer cells prefer glycolysis for energy production over oxidative phosphorylation, even under aerobic conditions [41]. Similarly, TASCs exhibit preferred metabolic patterns in pathological states, such as glycolysis in CAFs and TECs [42,43] and lipid metabolism in CAAs [44] (Fig. 3B). Cancer cells stimulate glycolysis of CAFs, which is referred to as the reverse Warburg effect. In this state, CAFs increase glucose uptake and lactate production [45]. Moreover, CAFs engage in glutamate/glutamine metabolism, compensating for the Warburg effect-induced suppression of glucose metabolism in tumor cells [46,47]. Similarly, enhanced glycolysis in TECs is advantageous for satisfying the oxygen demand for tumors [48]. Especially during angiogenesis, tip cells transform into a glycolytic phenotype via vascular endothelial growth factor A (VEGFA) to obtain sufficient adenosine triphosphate (ATP) [49]. Fructose-6-phosphate-2-kinase/fructose-2,6-bisphosphatase 3 (PFKFB3), an activator of glycolysis, has been demonstrated to drive metabolic dysregulation in TECs. Pharmacological blockade or inhibition of PFKFB3 could remodel the tumor vascular microenvironment, indicating potential therapeutic promise [50]. In parallel to glycolysis, lipid metabolism plays a central role. CAAs and lpCAFs fulfill malignant energy demands by releasing fatty acids, glycerol, and leptin [39,51]. Under the action of CAAs, lipid metabolism predominates in tumor cells. For example, CAAs up-regulated *HIF1A* via the IL-6 and Janus kinase (JAK)/signal transducer and activator of transcription 3 (STAT3) signaling pathways, rewiring the glucose metabolism of ovarian cancer cells to phosphatidylcholine synthesis [52].

The metabolic rewiring of the TME exerts far-reaching effects beyond energy provision, critically regulating tumor immune responses. In the context of immunosuppression, accumulated lactate inhibits the activity of cytotoxic T lymphocytes [53]. Indoleamine-2,3-dioxygenase (IDO), an up-regulated metabolic enzyme in CA-MSCs, establishes links between tryptophan metabolism and T cell exhaustion. This leads to tryptophan depletion and kynurenine accumulation, disrupting the normal metabolism of T cells [54].

More importantly, metabolic–epigenetic crosstalk is gaining considerable attention. Metabolites are essential substrates for epigenetic remodeling (e.g., acetylation and methylation). For instance, acetyl-coenzyme A derived from glycolysis drove tumor progression by enhancing histone acetylation [55]. Analogously, CAF-derived acetate mediated SP1 protein acetylation, up-regulating spermidine/spermine N1-acetyltransferase 1 (*SAT1*) expression to support pancreatic cancer growth [56]. S-Adenosyl methionine (SAM), as a substrate for nicotinamide N-methyltransferase (NNMT), participates in histone methylation. Research combining bladder cancer samples and in vivo experiments revealed that NNMT up-regulation in CAFs depleted SAM within the TME. Low levels of SAM led to restricted methylation and overexpression of serum amyloid A (SAA). SAA, in turn, promoted the infiltration of tumor-associated macrophages (TAMs), driving uroepithelial bladder cancer [57]. These findings underscore that the complex interplay between metabolic rewiring and epigenetic reprogramming drives tumor aggressiveness.

#### Tumor angiogenesis leads to deterioration

As the tumor enlarges, the tumor core becomes increasingly hypoxic and nutrient-deprived [58]. Angiogenesis is essential for overcoming this constraint. To initiate this process, TASCs release pro-angiogenic factors, such as VEGFA [59,60], platelet-derived growth factor (PDGF) [61], and CXCL12 [55], to recruit endothelial cell precursors and increase vascular permeability (Fig. 3C). Additionally, adipokines secreted by CAAs contribute to neovascularization in BC. These underlying mechanisms are involved in the activation of the interleukin-1 (IL-1) receptor pathway and Wnt/β-catenin signaling in endothelial cells [62,63].

In addition to angiogenesis, vascular co-option is an alternative survival strategy for tumors, whereby cancer cells co-opt the preexisting vasculature to obtain nutrients and support metastasis (Fig. 3C). This phenomenon has been extensively observed, such as in BC liver metastases and glioblastoma [64,65]. The presence of vascular co-option explains the limitations of anti-angiogenic therapy (AAT) and points to novel directions for therapeutic strategies.

### Tumor metastasis phase: Functional shift of TASCs

#### EMT facilitates invasion

During the entire course of carcinogenesis, EMT is vital for metastasis. Tumor cells undergoing EMT adopt a spindle-like morphology and acquire enhanced invasive abilities, which are conducive to extravasation [66] (Fig. 3D). TASC-derived secretions constitute the central mechanism of metastatic EMT. In a coculture experiment involving CAFs and BC cells, carcinoma cells in EMT states were characterized by increased expression of mesenchymal markers (e.g., N-cadherin and vimentin) and decreased expression of epithelial markers (e.g., E-cadherin and ZO-1) [67]. This phenotype shift can be pharmacologically reversed by inhibiting the transforming growth factor-β (TGF-β) pathway [68]. TGF-β, a dominant secretory factor, has been implicated in EMT activation across multiple malignancies. For example, all CAFs in colorectal cancer (CRC) [69], gastric cancer (GC) [70], and bladder cancer [71], TECs in hepatocellular carcinoma (HCC) [72], and CA-MSCs in melanoma [73] secrete TGF-β, thereby promoting EMT. Moreover, other factors, such as IL-6 derived from CAAs [36] and Semaphorin 3C from CAFs [74], trigger EMT during tumor invasion. Recently, more precise molecular mechanisms underlying EMT have been illuminated, revealing several specific functional subsets. Thrombospondin 2 (THBS2^+^) mCAFs, as a matrix CAF subset, were associated with EMT [75]. THBS2 accelerated EMT in lung adenocarcinoma by binding to syndecan-4 on tumor cells [76]. In addition, they secreted collagen type VIII α1 chain (COL8A1) to activate phosphoinositide 3-kinase (PI3K)–protein kinase B (AKT) signaling in malignant cells and drove EMT of CRC [77]. Complement factor D (CFD^+^) iCAFs, which were inflammatory CAFs found in metastatic CRC, accelerated EMT via the secreted frizzled-related protein 1 (SFRP1)–fibroblast growth factor receptor 2 (FGFR2)–HIF1 signaling axis [78].

#### ECM remodeling supports tumor metastasis

With respect to tumor metastasis, TASCs establish a favorable immune microenvironment through ECM remodeling, such as ECM deposition and ECM degradation [79] (Fig. 3E). In terms of deposition, myCAFs are the master architects of the ECM and secrete collagens, fibronectin, proteoglycans, and periostin [80,81]. Notably, heterogeneous CAF subtypes with unique collagen expression profiles have been proposed, which result in diverse ECM compositions and properties (Table 2). In addition to directly generating ECM constituents, CAFs secrete lysyl oxidase (LOX) and lysyl oxidase-like proteins (LOXLs) to mediate the cross-linking and maturation of collagen [82,83]. Dense components and robust structures ultimately catalyze matrix stiffening. Stiff ECMs subsequently affect cellular signaling pathways, ultimately exacerbating tumor progression [84]. For example, mechanical cues from rigid ECMs trigger transduction signals, such as Ras-homologous (Rho)/Rho-associated coiled-coil containing kinase (ROCK), which regulate the cytoskeleton and accelerate metastasis [85]. Moreover, the remodeled stroma acts as a native immune barrier, aiding tumor invasion. In PDAC, disturbed fiber orientations and increased collagen densities failed to mediate T cell migration into tumor islets [86]. Interestingly, periostin (POSTN^+^) CAFs, which were involved in ECM remodeling, suppressed TAMs via IL-6, establishing their dual role in tumor progression [87–89].

To degrade the ECM, CAFs secrete matrix metalloproteinases (MMPs) and urokinase-type plasminogen activators (uPAs) [90] (Fig. 3E). Similarly, CAAs participate in degradation by producing MMP2, MMP7, MMP9, MMP11, and plasminogen activator inhibitor-1 (PAI-1) [91]. Without intricate 3-dimensional (3D) structures, cancer cells are apt to undergo metastatic dissemination. Given that the ECM serves as a reservoir for cytokines, its degradation releases various growth factors such as TGF-β, VEGF, and FGF, which are instrumental in tumor angiogenesis and metastasis [92]. Moreover, ECM fragments act as chemotactic agents, accelerating tumor development. For instance, the collagen VI α3 chain attracted macrophages, facilitating EMT in BC [93]. Overall, the balance between ECM deposition and degradation is crucial for maintaining tissue homeostasis. Normalizing ECM remodeling or combining it with immunotherapy holds potential value as a new therapeutic direction [94].

## Spatial Decoding: Regional Specificity of TASCs

Recently, advances in spatial technologies have attached importance to spatial heterogeneity, including tumor internal ecological niches, cellular neighborhoods, and overall tumor spatial architecture. Spatial profiling of heterogeneity enables a novel biomarker framework for precise prognostic stratification and personalized immunotherapy optimization [95].

### Ecological niche in the TME: Distribution and interaction of TASCs

TASCs, epithelial cells, and immune cells collectively shape the cellular landscape and spatial architecture of tumors. Insights into the spatial structure of tumors elucidate the distribution and intercellular communication among these cells, offering novel perspectives on tumor growth, invasion, and metastasis.

#### Distributions and spatial heterogeneity of TASCs within the TME

**Fig. 4.**
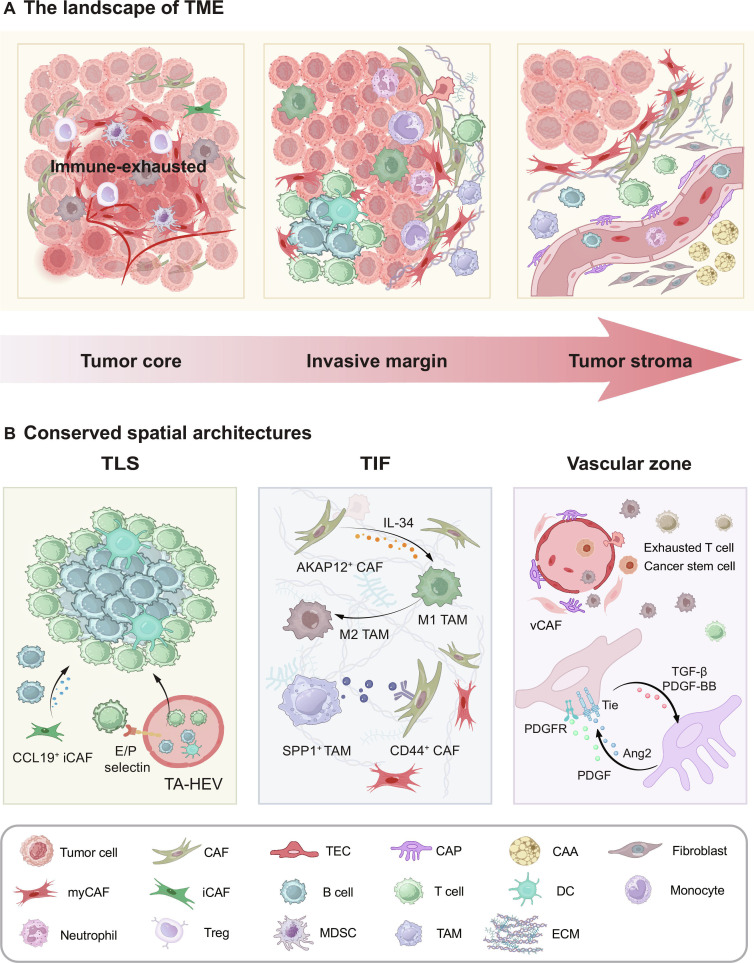
Spatial heterogeneity of TASCs in tumor niches. The overall landscape of TME and intertumoral ecological niches exhibit intricate complexity, with spatial heterogeneity of TASCs. (A) According to the anatomical location, TME is categorized into 3 main domains: tumor core, invasive margin, and tumor stroma. Based on this, TASCs exhibit heterogeneous spatial distributions and distinct cellular communications. Tumor core is immune-exhausted and enriched with inhibited immune cells as well as CAFs. The invasive margin refers to the dynamic interface where the tumor mass infiltrates into the adjacent normal tissue. Tumor stroma is populated by host cells (e.g., CAFs, CAAs, vessels, and various immune cells). (B) TASCs orchestrate key structural and functional niches critical to tumor progression. In TLS, iCAFs release CCL19 to attract B cells. TA-HEV, as an important component of TLS, provides entry for immune cells. The E/P selectin on TA-HEV is linked to T cell adhesion. In TIF, the close interactions between CAFs and macrophages reinforce immunosuppression. IL-34 derived from AKAP12^+^ CAFs induces M2 macrophage polarization. TAMs unleash SPP1, which binds to CD44 on CAFs and drives CAFs toward a profibrotic phenotype. The vascular zone is abundant with TECs, CAPs, vCAFs, exhausted T cells, and CSC. TGF-β and PDGF-BB from TECs recruit CAPs; in turn, CAPs maintain the relationship via Ang2/Tie and PDGF/PDGFR axis. AKAP12, A-kinase anchoring protein 12; CAAs, cancer-associated adipocytes; CAFs, cancer-associated fibroblasts; CCL19, C–C motif chemokine ligand 19; CD44, cluster of differentiation 44; CSC, cancer stem cell; DCs, dendritic cells; ECM, extracellular matrix; iCAFs, inflammatory cancer-associated fibroblasts; IL-34, interleukin-34; MDSCs, myeloid-derived suppressor cells; myCAFs, myofibroblastic cancer-associated fibroblasts; PDGF, platelet-derived growth factor; PDGF-BB, platelet-derived growth factor BB; PDGFR, platelet-derived growth factor receptor; SPP1, secreted phosphoprotein 1; TAMs, tumor-associated macrophages; TASCs, tumor-associated stromal cells; TA-HEV, tumor-associated high endothelial venules; TECs, tumor-associated endothelial cells; TGF-β, transforming growth factor-β; TIF, tumor invasive front; TLS, tertiary lymphoid structure; TME, tumor microenvironment; Tregs, regulatory T cells; vCAFs, vascular cancer-associated fibroblasts.

From the perspective of the TME, spatial heterogeneity is characterized by 3 functionally distinct compartments: the tumor core, the invasive margin, and the tumor stroma [96]. The characteristics of the cellular composition within each compartment are delineated in Fig. 4A. The tumor core, as an immune-exhausted zone, is dominated by tumor cells, immunosuppressive leukocytes [e.g., TAMs, myeloid-derived suppressor cells (MDSCs), and regulatory T cells (Tregs)], as well as a few CAFs and TECs [96]. The invasive margin serves as a 2-sided battlefield for immune surveillance and tumor escape. The dynamic balance between protumor factors (e.g., CAFs and M2 macrophages) and antitumor factors [e.g., tertiary lymphoid structures (TLSs), CD8^+^ T cells, and natural killer (NK) cells] in this region makes it critical for metastasis [97]. The outermost stromal region of the tumor constitutes a supportive microenvironment, composed of CAFs, CAAs, the ECM, and blood vessels [98].

The spatial heterogeneity of TASCs affects their functions and interactions with other cells. Here, we delve into the distribution of CAFs and TECs from the perspective of the overall TME. The unique niches of major CAF subsets have been revealed with spatial mapping. myCAFs are in proximity to tumor cells and are concentrated within the stromal compartment or the invasive front [99,100]. vCAFs are enriched in the vascular zone [101], and apCAFs colocalize with immune cells such as macrophages and T cells, resulting in synergistic immunological effects [102]. In contrast to the peritumoral localization of myCAFs, iCAFs are located in fibrogenic areas distant from tumor cells according to the initial definition [6,103]. The spatial resolution of multiple tumors has further confirmed this phenomenon, suggesting that the differences in tumor origins and activated signals are potential drivers of spatial heterogeneity [104]. Interestingly, defined by their enrichment in hypoxic regions, hypoxic CAFs (HCAFs) exhibit a stronger correlation with hypoxia levels than other CAFs [105].

TECs are distributed in the tumor core and margins along with the vascular system. Compared with peritumoral endothelial cells, tumor-core TECs exhibit marked heterogeneity in their abundances, molecular subtypes, and function. First, the overall abundance of TECs is markedly increased along with a rise in pericyte abundance [106]. Second, the proportions of TEC subtypes are dysregulated, with a reduction in capillary endothelial cells and an enrichment of immature TECs and pro-angiogenic tip cell subtypes [107]. These changes reflect profound dysregulation of vascular maturation. Third, the TEC-enriched signaling pathways in the core region are biased toward angiogenesis. According to scRNA-seq analysis of glioblastoma patients, TECs in the tumor core acquired genetic signatures linked to basement membrane remodeling and tip cell formation. This subpopulation was predominantly clustered in the microvascular proliferation (MVP) region, which functionally manifested enhanced angiogenic capacity [108,109]. TECs also exhibited impaired immune defense functions, as evidenced by the suppressed expression of interferon-stimulated genes and the transcriptional program for antigen presentation (pS05) in CRC [110]. Furthermore, TECs actively enforced immune exclusion via FasL-mediated CD8^+^ T cell exhaustion and secretion of CXCL12/14, inhibiting cytotoxic T cell maturation [111–113].

While TECs in the tumor core possess a highly abnormal and immunosuppressive phenotype, their counterparts at the tumor margin present a distinct, yet equally consequential niche. The tumor margins are more densely packed with relatively normal functioning vessels and a higher percentage of venous endothelium. For example, a venous microenvironment consisting of atypical chemokine receptor 1-positive (ACKR1^+^) TECs and C–C motif chemokine ligand 21-positive (CCL21^+^) peripheral pericytes was present in the tumor margin area and metastatic lymph nodes in BC. ACKR1^+^ TECs were enriched in postcapillary microvessels, whereas CCL21^+^ pericytes formed concentric laminar structures around these vessels. These cells spatially synergized to form a chemokine-rich microenvironment that promoted the infiltration of C–C motif chemokine receptor 7-positive (CCR7^+^) lymphocytes, highlighting the critical role of spatial heterogeneity in immunotherapy [114].

#### Spatial architectures composed of TASCs

Specific aggregations of TASCs with surrounding cells constitute unique ecological niches. These niches are not only landmarks of tumors’ spatial architectures but also functional units that shape the functional heterogeneity of the TME. Several niches containing the immune-related TLS, tumor-invasive front (TIF), and vascular zone have been extensively characterized (Fig. 4B).

TLSs, as ectopic lymphoid organs, are predominantly found at the invasive margin or stromal regions of tumors [115]. The main components include immune protective cells [e.g., B cells, T cells, and dendritic cells (DCs)] and tumor-associated high endothelial venules (TA-HEVs) [116]. To recruit constituents and orchestrate spatial architectures, TASCs function as organizers. CAFs maintain lymphocyte aggregates by expressing lymphotoxin β receptor (LTβR) and tumor necrosis factor receptor (TNFR) [117]. Specifically, CCL19^+^ iCAFs were confirmed to recruit B cells through the CCL19/CCR7 axis in a humanized CRC model [118]. TA-HEVs, a specialized subset of TECs, provide convenient entries for immune cell infiltration [119]. In vitro experiments of nasopharyngeal carcinoma have demonstrated that TA-HEVs were induced by type I and type II interferons. These TA-HEVs, in turn, mediated immune responses through colocalization with CD4^+^ T cells via CXCL9 [120]. Similarly, TA-HEVs enhanced the attraction, adhesion, and infiltration of CD8^+^ T cells by expressing sialomucins and E/P-selectins [121]. Following insights into the components and forming mechanisms, TLSs have been established as an important marker of good prognoses, which was first revealed in non-small cell lung carcinoma (NSCLC) [116]. Building on this foundation, deepening the knowledge of TASCs in TLSs, especially TA-HEVs, has paved the way for new therapeutic avenues in modulating the tumor immune microenvironment (TIME).

In contrast to the immune protection of TLSs, TIF, a spatially and dynamically heterogeneous region, orchestrates local invasion and distant metastasis by counteracting immune activities [122]. With the advancement of ST, the landscape of TIF has become clear. Zhou’s team [123] achieved subcellular-level localization of TIF in HCC using Stereo-seq, a new ultrahigh-resolution technology. This invasive zone was defined as the special band spanning 500 μm on the transition area of carcinoma and paracarcinoma tissue. Technologies have further characterized the profound immunosuppression within the TIF, shedding light on the critical crosstalk between CAFs and macrophages [124]. On the basis of CellPhoneDB and ST analyses, the spatial proximity of A-kinase anchoring protein 12 (AKAP12^+^) CAFs and M2 macrophages in triple-negative breast cancer (TNBC) suggested that CAFs induced macrophage polarization via interleukin-34 (IL-34). IL-34 signaling blockade with programmed cell death-1 (PD-1) therapy promoted immunotherapeutic efficacy in vivo experiments [125]. Moreover, TAMs and CAFs cooperate to construct immune barriers around tumor boundaries. Secreted phosphoprotein 1 (SPP1) from TAMs, as a potent mediator of fibrosis, enhances the profibrotic function of CAFs through combination with a cluster of differentiation 44 (CD44) [126]. The fibrotic barrier, formed by CAFs, hinders T cell infiltration into the tumor core [127]. In contrast, in BC, myCAFs promoted CD8^+^ T cell aggregation through the expression of elastin microfibril interfacer 1 (EMILIN1), which inactivated TGF-β signaling. This finding contradicted the previously held perception of myCAFs as uniformly immunosuppressive [128].

In addition to the above 2 niches, the vascular zone establishes a pathway for tumor cell dissemination. TECs line the inner side of blood vessels [129], whereas CAPs reside on the exterior and are embedded in the basement membrane [130]. In addition, sparse vCAFs encircle the vasculature, acting as critical mediators of angiogenesis [101]. Close spatial apposition and interaction between TECs and CAPs are essential for maintaining vascular homeostasis. Cytokines secreted by TECs, such as PDGF-BB and TGF-β, are crucial mediators of CAP recruitment [131]. In turn, CAPs maintain the quiescent state of TECs through angiopoietin-2 (Ang2)/Tie and PDGF/platelet-derived growth factor receptor (PDGFR) [132,133]. However, in response to the TME, this cooperative relationship is disrupted. CAPs detach from endothelial cells, leading to anomalous vascular structures that facilitate cancer cell metastasis [132]. For example, elevated prostaglandin E2 (PGE2) levels in the TME impaired TEC-CAP connections by down-regulating N-cadherin expression in CAPs [134]. Furthermore, dysregulated pericyte contractility contributed to poor vascular perfusion and vessel leakiness. This characteristic was mechanistically linked to the expression of regulator of G protein signaling 5 (RGS5) [135].

TECs also organize the perivascular microenvironment through interactions with immune cells and cancer stem cells (CSCs). For example, TECs induced the accumulation of M2 macrophages and the depletion of cytotoxic T cells, thereby establishing an immunosuppressive milieu [136,137]. Concurrently, TECs cultivated a fertile ground for CSCs, especially in brain tumors, where their co-occurrence was critical for tumor stemness [138,139]. Another study demonstrated that TECs promoted the malignant transition of glioma stem cell-like cells through the MMP–nuclear factor κB (NF-κB) pathway [140].

### Heterogeneous landscape of the tumor core: The spatial heterogeneity of TASCs between primary and metastatic tumors

The malignant evolution of tumors is a highly coordinated spatial process. From primary sites to distal organs, stromal components of the TME display spatial heterogeneity. In primary sites of GC, stromal cells, including CAFs, TECs, and protective immune cells (e.g., DCs and cytotoxic T cells), constituted a relatively well-balanced, yet perturbed microenvironment. In contrast, peritoneal metastases were marked by exhausted CD8^+^ T cells [141]. Single-cell analyses of metastatic samples from other malignancies further confirm a trend toward reduced stromal infiltration and enhanced immunosuppression of metastatic foci. For instance, the proportion of CAFs was reduced in pancreatic cancer liver metastases [142], and a marked increase in M2 macrophages was detected in lung adenocarcinoma brain metastases [143]. Moreover, CAF-derived insulin-like growth factor-binding protein 2 (IGFBP2) in peritoneal metastatic sites of CRC inhibited macrophage activation and T cell proliferation [144].

The pronounced functional reprogramming of TASCs in metastatic lesions further shapes spatial heterogeneity. Before the arrival of tumor cells, the primary tumor remotely modifies TASCs in distant organs to establish a premetastatic microenvironment (PMN) [145]. CXCL14 from osteosarcoma stimulated CAFs to secrete TGF-β, thereby attracting tumor cells to the lung niche [146]. Following dissemination, TASCs facilitate tumor cell lodging. Melanoma cell-derived extracellular vesicles (EVs) in murine models promoted the adhesion of tumor cells to the endothelium by up-regulating intercellular adhesion molecule 1 (ICAM1) expression on TECs [147]. Lymphatic TECs supported metastasis through stromal cell-derived factor-1 (SDF-1) binding to the CXCR4 on tumor cells [148]. Lastly, unique cell groups are enriched in metastases. Single-cell analysis of 4 resected esophageal squamous cell carcinoma (ESCC) specimens revealed increased numbers of pericytes with angiogenic features in metastatic lymph nodes, whereas in the primary foci, their counterparts predominantly regulated cell migration. A distinct pericyte subset (C6_pericyte), marked by high expression of Thy-1 cell surface antigen (THY1), PDGFRβ, RGS5, and NDUFA4 mitochondrial complex-associated like 2 (NDUFA4L2), was enriched in metastatic lymph nodes. This subset was proposed to orchestrate the pericyte–fibroblast transition (PFT) to accelerate tumor progression [149].

### A comparative view of the distribution of TASCs across tumor types

Different tumor types display a preference for specific target organs, shaping the spatial heterogeneity of TASCs at the organ level. CAFs demonstrate conserved and context-dependent heterogeneity across solid tumors (Table 3). While myCAFs are ubiquitous in desmoplastic tumors (e.g., PDAC and BC), the abundance of iCAFs increases in aggressive and immunosuppressive niches [150]. Crucially, iCAF subpopulations drive divergent immune landscapes. In “hot” tumors, iCAFs interact with B cells and T cells via the CXCL12–CXCR4 axis, whereas in “cold” tumors, which are infiltrated by MDSCs and Tregs, such as prostate cancer and liver cancer, iCAFs are deficient [151].

**Table 3.** TASC subtypes across tumors

Cancer type	Model	Subtype		Biomarker	Subtype function	Spatial pattern	Refs.
*CAF heterogeneity*							
Bladder cancer	Human	PDGFRA^+^ iCAFs		*CXCL12*, *Ly6c1^high^*	Chemokines and cytokine secretion	NA	[261]
		RGS5^+^ myCAFs		RGS5	Muscle contraction and ECM remodeling	NA	
Breast cancer	Human	LRRC15^+^ myCAFs		*LRRC15*, *COL10A1*, *MMP11*	Associated with immunosuppression and tumor progression	Enriched in the tumor core, adjacent to tumor cells	[19]
	Human and murine models	PDPN^+^ CAFs		*PDPN*	Secreting inflammatory factors (CXCL12, IL-6), promoting wound healing, and ECM remodeling	Adjacent to tumor cells	[226]
		S100A4^+^ CAFs		*S100A4*	Antigen presentation, ECM remodeling	Distributed in the dense matrix region	
	MMTV-PyMT mouse model	Vascular CAFs		*NOTCH3*, *EPAS1*, *COL18A1*, *NR2F2*	Angiogenesis, basement membrane remodeling	Perivascular location	[10]
		Matrix CAFs		*FBLN1*, *FBLN2*, *COL14A1*, *PDGFRΑ*	Related to ECM	NA	
		Developmental CAFs		*SCRG1*, *SOX9*, *SOX10*	Involving in the EMT transformation of malignant cells	NA	
		Cycling CAFs		*Ki-67*	Related to cell sorting as the dividing state of vCAFs	NA	
	Human	IL-iCAFs		*GPC3*, *DLK1*	Involving in the interleukin signaling pathway	NA	[100]
		IFN-γ-iCAFs		*ISG15*, *IFITM1*, *STAT1*, *IRF*	Antigen presentation, inhibiting TGF-β signaling and attracting CD8^+^ T cells	NA	
		Detox-iCAFs		*GPC3*, *DLK1*	Immune protection	Mainly distributed around the tumor, close to blood vessels	
		ECM-myCAFs		*SDC1*, *COL1A2*	Involving in tumor stroma remodeling	Enriched in tumors	
		TGF-β-myCAFs		*LAMP5*	Associated with the TGF-β signaling pathway, suppresses the immune	Colocalized with TREM2^+^ macrophages and regulatory T cells	
		Wound-myCAFs		NA	Wound healing	Located in the intratumor stroma, away from the cancer cells	
		Acto-myCAFs		*ACTA2*	Related to the actin pathway	NA	
		IFN-α/β-myCAFs		NA	Immunomodulation through the interferon αβ signaling pathway	NA	
	MMTV-PyMT mouse model	CD63^+^ CAFs		*CD63*	Advanced chemotherapy resistance	NA	[262]
Carcinoma of the colon and rectum	Human	MMP1^+^ myCAFs		*MMP1*, *CXCL1*, *CCL11*	Pro-inflammatory and immunosuppressive function	Colocalized with Tregs and LAMP3^+^ dendritic cells	[19]
	Human	Contractile CAFs		*RGS5*, *MCAM*, *MYH11*	ECM remodeling, angiogenesis	Enriched in the tumor metastatic junction area	[263]
		ECM-remodeling CAFs		*LTBP2*, *C3*, *POSTN*, *MMP2*	ECM remodeling	Gathered in regions with pronounced desmoplastic response	
	Human and murine models	iCAFs		*IL1A*, *IL1B*, *IL6*, *CXCL1*, *CXCL5*	Accelerating metastasis by neutrophil extracellular trap	NA	[237]
Hepatocellular carcinoma	Human	CAFs		*CD36*, *POSTN*, *STMN1*, *CXC12*, *SEPT7*, *MYH11*	POSTN^+^ CAFs repelled T cells and recruited TAM	Colocalized with SPP1^+^ macrophages	[88]
	Human	CCL19^+^ CAFs		*CCL19*, *CCL21*, *C7*, *CFH*, *C1S*	Driving T cell migration and leukocyte migration	Colocalized with plasma cells	[264]
Lung cancer	Human and murine models	apCAFs		*CD74*, *SLPI*, *IL6*, *CFD*, *C1QA*, *C1QB*	Immune protection	NA	[265]
	Human	Subtype I		*HGF^high^*, *FGF7^high/low^*, *p-SMAD2^low^*	Protection and promotion of cancer cell proliferation	Enriched in the invasive front of the primary tumor	[266]
		Subtype II		*HGF^low^*, *FGF7^high^*, *p-SMAD2^low^*	Medium protection	Distributed in the tumor core and colocalized with vascular areas	
		Subtype III		*HGF^low^*, *FGF^/low^*, *p-SMAD2^high^*	High expression of chemokines, promoting infiltration of CD8^+^ T cells and monocytes	Enriched in the tumor margin or fibrotic mesenchyme	
Ovary cancer	Human	CAFs		*CD29*, *FAP*, *α-SMA*, *FSP1*, *PDGFRβ*, *CAV1*	Immunomodulation and ECM-related	NA	[267]
Pancreatic ductal adenocarcinoma	Human and murine models	myCAFs		*NDUFA4L2*, *ACTA2*, *MYL9*, *α-SMA^high^*, *FAP*	Extracellular matrix remodeling and fibrosis	NA	[7]
		iCAFs		*IL-6*, *IL-11*, *α-SMA^low^*, *PDGFRα^high^*, *CXCL12*	Immune modulation	NA	
		apCAFs		*CD74*, *H2-Ab1*, *HLA-DRA*	Antigen-presenting with MHC class II molecules	NA	
	Genetically engineered mouse models (GEMMs)	Lipid-laden CAFs		*ABCA8a*	Lipid metabolism supported tumor growth	NA	[39]
	Human	Metabolic CAFs		*PLA2G2A*, *CRABP2*, *LDHA*, *PKM2*	Implications of poor prognosis and promising immunotherapeutic effects	NA	[268]
	Human	Interferon-response CAFs		*RSAD2*, *IFIT3*, *CXCL10*	Inhibiting tumor invasion and inducing neutrophils; STING agonists accelerated the transformation	NA	[269]
	Murine models	FAPα^+^CD144^+^ endothelial-like CAFs		*FAPα*, *CD144*	Promoting tumor invasion by vasculogenic mimicry	NA	[270]
Pan-cancer	Human	S1-CAFs		*ACTA2*, *TGFB1*, *COL1A1*/*3A1*, *MMP11*	Consisting immune barrier	Adjacent to tumor cells	[221]
		S2-CAFs		*IL-6*, *LIF*, *FAP*, *PDPN*, *CXCL12*	Interaction with endothelial cells	Located in the stroma	
		S3-CAFs		*PDGFRB*, *CXCL14*	Crosstalk with myeloid cells	Distributed in myeloid cell aggregation	
		S4-CAFs		*CCL19/21 HLA-II*	Recruiting lymphocytes	TLS	
Prostate cancer	Human	Cluster 0		*GLRX*, *CCL2*	Immunosuppressive microenvironment	Located at the tumor–stromal junction or around blood vessels, forming immunosuppressive microenvironments	[271]
		Cluster 1		*PKM*, *LRP1*, *CXCL12*	Recruiting immune cells via CXCL12	NA	
		Cluster 2		*CD63*	Secreting ECM proteins (e.g., collagen)	Distributed in ECM-rich regions	
		Cluster 3		*BIRC5*	Inhibiting apoptosis and promotes cell proliferation	Proximity to tumor cell nests	
		Cluster 4		*PHKG1*	Metabolic reprogramming	Located in hypoxic or metabolically active areas	
		Cluster 5		*MALAT1*	Epigenetic regulation influenced tumor progression	Distributed in ECM-rich regions	
*TEC heterogeneity*							
Breast cancer	Human	LIPEC		*ABP4*, *CD36*, *PPARγ*, *LXRA*	Involving lipid uptake, storage, and metabolism	NA	[15]
		Immunomodulation ECs		*HLA-DRA*	Modulating the immune microenvironment	NA	
		Pro-angiogenic ECs		*APLNR*, *KDR*, *COL4A1*	Angiogenesis	NA	
Colorectal cancer	Human	C1_TECs		*ESM1*, *NID2*	Acting as Tip cells, promoting vascular outgrowth	Resided in malignant tissue, adjacent to tumor cells	[272]
		C2_TECs		*ACKR1*, *SELP*	Acting as HEVs/vein endothelium, involving lymphocyte homing	Enriched in tumors	
		C3_TECs		*CA4*, *CD36*	Capillary endothelium	NA	
		C4_TECs		*FBLN5*, *GJA5*	Arterial endothelial cell	NA	
		C5_TECs		*PROX1*, *PDPN*	Acting as lymphatic endothelium, supporting lymphangiogenesis	NA	
Head and neck squamous cell carcinoma	Human	Lymphatic ECs		*YVE1*, *PROX1*, *PDPN*, *FLT4*	Involving lymphatic vessel formation and immune cell transport	Distributed at tumor margins or stromal areas, adjacent to areas of immune cell infiltration	[273]
		Vascular ECs	Pre-capillary endothelial cells	*ACKR1*, *PLVAP*	Angiogenesis, immune cell infiltration	Resided in the frontiers of tumor invasion	
			Posterior capillary endothelial cells	*CD34*, *VWF*		Surrounded by mature blood vessels	
			Active endothelial cells	*SELE*, *ICAM1*		Resided in the high-inflammation area	
Lung cancer	Human and murine models	Arterial ECs		*EFNB2*, *GJA5*, *DLL4*	Maintaining vascular integrity	Surrounded by the aorta and resistance arteries	[107]
		Venous ECs		*ACKR1*, *NR2F2*	Leukocyte recruitment	Surrounded by the veins and post-capillary microvessels	
		Capillary ECs		*EMCN*, *VWF*, *CD36*	Substance exchange, antigen presentation	NA	
		Tip ECs		*CXCR4*, *PGF*, *ANGPT2*	Inducing vascular sprouting	Resided in the frontiers of vascular neovascularization	
		HEV-like ECs		*CCL21*, *SELP*	Immune cell infiltration	NA	
		Lymphatic ECs		*PROX1*, *LYVE1*	Lymphatic drainage, immunosurveillance	NA	
Pan-cancer	Human	Tip cells		*KDR*, *CXCR4*, *DNAJB1*, *ATP5E*, *STMN1*	Involving angiogenesis, stress response, ATP synthesis, and cell cycle	CXCR4^+^ tip cells were located in the tumor core, colocalized with epithelial or malignant cells	[106]
		Veins		*SELE*, *CLU*, *FABP4*, *COL4A1*, *IGFBP5*	Pro-inflammatory, collagen formation, lipid metabolism	SELE-Veins were located at the tumor periphery, spatially close to T cells	
		Capillaries		*TMEM100*, *FABP4*, *RGCC*	Capillary function, antigen presentation	NA	
		Arteries		*DNAJB1*, *CLU*, *COL1A1*	Arterial function, collagen formation	NA	
		Lymphatics		*PROX1*, *LYVE1*	Lymphatic function	Located in the lymphatic regions	
		Hypoxia-associated endothelial cells		*MT1X*, *MT1E*, *MT2A*	Hypoxia response	Located at the tumor periphery, spatially close to T cells	

α-SMA, α-smooth muscle actin; ABCA8a, ATP-binding cassette subfamily A member 8a; ABP4, adipocyte-binding protein 4; ACKR1, atypical chemokine receptor 1; ACTA2, actin α2, smooth muscle; ANGPT2, angiopoietin-2; APLNR, apelin receptor; ATP5E, ATP synthase F1 subunit epsilon; BIRC5, baculoviral IAP repeat-containing 5; C, complement; CAFs, cancer-associated fibroblasts; CAV1, caveolin-1; CCL, C–C motif chemokine; CCR5, C–C motif chemokine receptor 5; CD, cluster of differentiation; CFD, complement factor D; CFH, complement factor H; COL, collagen type; CXCL, C–X–C motif chemokine; CXCR4, C–X–C motif chemokine receptor 4; DLK1, delta-like noncanonical Notch ligand 1; DLL4, delta like canonical Notch ligand 4; DNAJB1, DnaJ heat shock protein family (Hsp40) member B1; ECM, extracellular matrix; EFNB2, ephrin B2; EMCN, endomucin; EMT, epithelial–mesenchymal transition; ESM1, endothelial cell-specific molecule 1; FABP4, fatty acid-binding protein 4; FAP, fibroblast activation protein; FBLN1, fibulin 1; FBLN2, fibulin 2; FGF7, fibroblast growth factor 7; FLT4, Fms-related receptor tyrosine kinase 4; FSP1, fibroblast-specific protein 1; GJA5, gap junction protein α5; GLNX, glutaredoxin; GPC3, glypican 3; HEVs, high endothelial venules; HGF, hepatocyte growth factor; HLA-DRA, major histocompatibility complex, class II, DRα; HLA-II, human leukocyte antigen class II; ICAM1, intercellular adhesion molecule 1; IFIT3, interferon induced protein with tetratricopeptide repeats 3; IFITM1, interferon induced transmembrane protein 1; IFN, interferon; IGFBP5, insulin like growth factor binding protein 5; IL, interleukin; IRF, interferon regulatory factor; ISG15, ISG15 ubiquitin-like modifier; KDR, kinase insert domain receptor; LAMP5, lysosomal-associated membrane protein family member 5; LDHA, lactate dehydrogenase A; LIF, leukemia inhibitory factor; LIPEC, lipid-processing endothelial cells; LRRC15, leucine rich repeat containing 15; LRP1, LDL receptor related protein 1; LTBP2, latent transforming growth factor β binding protein 2; LXRA, liver X receptor α; LYVE1, lymphatic vessel endothelial hyaluronan receptor 1; MALAT1, metastasis associated lung adenocarcinoma transcript 1; MCAM, melanoma cell adhesion molecule; MHC, major histocompatibility complex; MMP, matrix metallopeptidase; MMTV-PyMT, mouse mammary tumor virus-polyoma middle T-antigen; MT, metallothionein; MYH11, myosin heavy chain 11; MYL9, myosin light chain 9; NA, not applicable; NDUFA4L2, NDUFA4 mitochondrial complex associated like 2; NID2, nidogen 2; NOTCH3, notch receptor 3; NR2F2, nuclear receptor subfamily 2 group F member 2; PDPN, podoplanin; PDGFR, platelet-derived growth factor receptor; PHKG1, phosphorylase kinase catalytic subunit γ1; PGF, placental growth factor; PKM, pyruvate kinase M1/2; PLA2G2A, phospholipase A2 group IIA; PLVAP, plasmalemma vesicle-associated protein; PPARγ, peroxisome proliferator-activated receptor γ; PROX1, prospero homeobox 1; RAMP3, receptor activity modifying protein 3; RGS5, regulator of G protein signaling 5; RGCC, regulator of cell cycle; RSAD2, radical S-adenosyl methionine domain containing 2; S100A4, S100 calcium-binding protein A4; SCRG1, stimulator of chondrogenesis 1; SDC1, syndecan 1; SELP, P-selectin; SELE, E-selectin; SEPT7, septin 7; SLPI, secretory leukocyte peptidase inhibitor; SMAD2, mothers against decapentaplegic homolog 2; SOX10, SRY-box transcription factor 10; SOX9, SRY-box transcription factor 9; SPP1, secreted phosphoprotein 1; STAT1, signal transducer and activator of transcription 1; STING, stimulator of interferon genes; STMN1, stathmin 1; TAMs, tumor-associated macrophages; TECs, tumor-associated endothelial cells; TGF-β, transforming growth factor-β; TGFB1, transforming growth factor-β1; TREG, regulatory T cell; TSPAN8, tetraspanin 8; VWF, von Willebrand factor

Single-cell mapping of TECs across cancer types, such as lung, liver, and brain malignancies, has revealed intertumor heterogeneity and dynamic plasticity [16,106]. Human scRNA-seq data have indicated that TECs in glioblastoma up-regulate plasma lemma vesicle-associated protein (PLVAP) for transcellular transport but down-regulate transporter protein genes [e.g., ATP binding cassette subfamily B member 1 (ABCB1) and ATP binding cassette subfamily G member 2 (ABCG2)], suggesting compromised blood–brain barrier integrity [108]. In HCC, TECs lack the expression of canonical liver-sinusoidal endothelial markers, C-type lectin domain family 4 member G (*CLEC4G*), and express macrovascular endothelial signatures [e.g., platelet and endothelial cell adhesion molecule 1 (*PECAM1*), aquaporin 1 (*AQP1*), and *CD34*] as well as PLVAP. PLVAP induces a decrease in endothelial permeability, which in turn mediates immune escape, shaping the immunosuppressive microenvironment [152]. In fat-infiltrated cancers, particularly BC and ovarian cancer, adipocytes constitute a substantial portion of the tumor stroma, promoting inflammation, metabolic rewiring, and cancer cell modulation [153–155]. The differentiation potential of CA-MSCs into CAFs, TECs, and adipocytes may vary across different tumors, leading to diverse functional mechanisms [156,157]. This variability necessitates further research to elucidate the specific roles and mechanisms of CA-MSCs in various tumor contexts.

## Immune Escape Resolution: Dynamic Association of TASCs with Immune Checkpoints

Immune checkpoints are molecules that prevent immune overactivation in the physiological state, such as PD-1, programmed death-ligand 1 (PD-L1), and cytotoxic T lymphocyte-associated antigen-4 (CTLA-4). However, tumors exploit these checkpoints to evade immune detection, and inhibitors are used to enhance antitumor immunity [158]. In this context, understanding the regulatory relationships between TASCs and immune checkpoints is necessary.

**Fig. 5.**
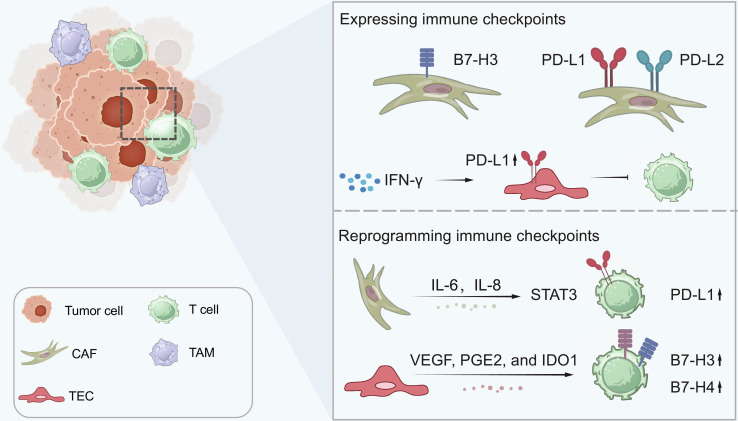
Interactions between TASCs and immune cells. TASCs interplay with immunosuppression by orchestrating immune checkpoints. TASCs could express related proteins, like B7-H3, PD-L1, and PD-L2, on CAFs. Moreover, in response to IFN-γ, TECs up-regulate PD-L1, thereby further suppressing T cell activity. The panel below demonstrates the TASC-driven up-regulation of immune checkpoints on immune cells. IL-6 and IL-8 from CAFs trigger the STAT3 signaling in T cells, promoting PD-L1 expression. TECs also augment the expression of B7-H3 and B7-H4 on T cells via VEGF, PGE2, and IDO1. B7-H3, B7 homolog 3; CTLA-4, cytotoxic T lymphocyte-associated protein 4; IDO1, indoleamine 2,3-dioxygenase 1; IFN-γ, interferon-γ; IL-6, interleukin-6; IL-8, interleukin-8; PGE2, prostaglandin E2; PD-L1, programmed death-ligand 1; PD-L2, programmed death-ligand 2; TAMs, tumor-associated macrophages; TASCs, tumor-associated stromal cells; VEGF, vascular endothelial growth factor.

TASCs are involved in the regulation of immune checkpoints through an array of mechanisms. We systematically summarized the 2 main ways: expressing immune checkpoint ligands and orchestrating the immune checkpoints on other cells through cytokines (Fig. 5). First, TASCs themselves express a range of ligands that interact with immune checkpoints. PD-L1/PD-L2 [159,160] and B7 homolog 3 (B7-H3) [161] are present on the surface of CAFs. TECs express PD-L1, which is induced by interferon-γ (IFN-γ). In vivo experiments demonstrated that PD-L1^+^ TECs blunted the activation and proliferation of CD8^+^ T cells [162]. PD-L1 has also been detected in murine brown adipose tissue, suggesting a potential link between CAAs from this tissue and PD-L1 expression [163]. Next, TASCs reprogram checkpoints on immune cells, disrupting immune defense. Analyses of human samples have unveiled that the abundance of CAFs is correlated with an enhanced CTLA-4 gene signature in CD8^+^ T cells, which accounts for the T cell exclusion [164]. CAFs connect with PD-1/PD-L1 signaling on T cells through the secretion of IL-6 [165] and IL-8 [166,167] to trigger the STAT3 pathway. In parallel, TECs up-regulate the expression of PD-L1, B7-H3, and B7 homolog 4 (B7-H4) on T cells via factors such as VEGF, PGE2, and IDO1 [168,169].

## Reprogramming of TASCs: An Emerging Paradigm in Cancer Immunotherapy

**Fig. 6.**
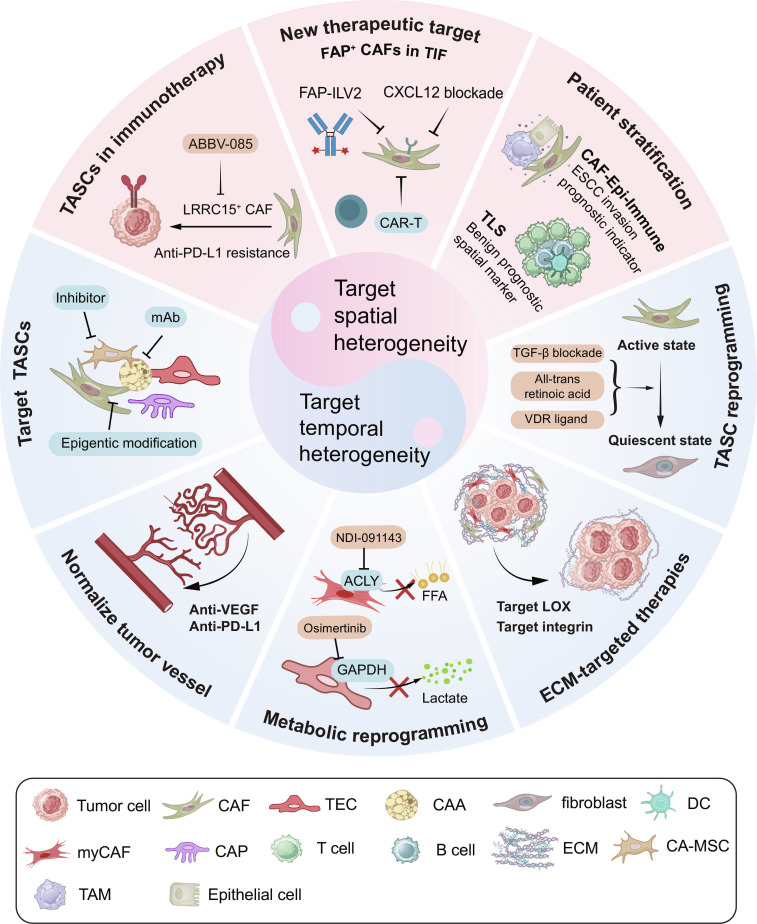
Therapies targeting TASCs in the context of spatiotemporal heterogeneity. The spatiotemporal heterogeneity of TASCs dynamically shapes the tumor immune microenvironment, which provides a theoretical basis for the development of novel immunotherapeutic targets. The following outlines specific strategies for targeting TASCs to remodel the TME. TASCs in immunotherapy: LRRC15^+^ CAFs, which drive resistance to anti-PD-L1 therapy, could be selectively eliminated with agents ABBV-085 to restore treatment sensitivity. New therapeutic targets, such as FAP^+^ CAFs within the TIF: To counteract their pro-invasive role, several strategies are under development, including FAP-ILV2, blockade of the activation signal (CXCL12), and CAR-T cells targeting FAP. Patient stratification: The structural analysis of the tumor ecosystem is vital for staging, prognostication, and identifying novel therapeutic targets. For example, the presence of TLS is a favorable prognostic indicator. Conversely, the CAF-Epi-Immune niche, a unique structure identified in esophageal cancer, is composed of CAFs, epithelial cells, and immune cells and serves as a marker of tumor invasion. TASCs reprogramming: reverting active TASCs into a quiescent state. Effective agents for CAF reprogramming include TGF-β inhibitors, all-trans retinoic acid, and VDR ligands. ECM-target therapies: degrading this physical barrier to enhance immune cell infiltration. Promising targets include LOX and integrins. Metabolic reprogramming: remodeling TASC metabolism to alleviate immunosuppression. For example, NDI-091143 inhibits ACLY in CAFs, reducing the energy supply of FFA. Osimertinib suppresses GAPDH in TECs, reducing lactate production. Targeting TASCs: utilizing inhibitors or monoclonal antibodies against surface markers, or applying epigenetic reprogramming to reverse protumor phenotypes. ACLY, ATP citrate lyase; CA-MSCs, carcinoma-associated mesenchymal stem cells; CAFs, cancer-associated fibroblasts; CAAs, cancer-associated adipocytes; CAPs, cancer-associated pericytes; CAR-T, chimeric antigen receptor T cell; CXCL12, C–X–C motif chemokine ligand 12; DCs, dendritic cells; ECM, extracellular matrix; ESCC, esophageal squamous cell carcinoma; FAP, fibroblast activation protein; FAP-ILV2, fibroblast activation protein-interleukin-2 variant; FFA, free fatty acid; GAPDH, glyceraldehyde-3-phosphate dehydrogenase; LOX, lysyl oxidase; LRRC15, leucine-rich repeat-containing 15; mAb, monoclonal antibody; myCAFs, myofibroblastic cancer-associated fibroblasts; PD-L1, programmed death-ligand 1; TAMs, tumor-associated macrophages; TASCs, tumor-associated stromal cells; TECs, tumor-associated endothelial cells; TGF-β, transforming growth factor-β; TIF, tumor invasion front; TLS, tertiary lymphoid structure; VDR, vitamin D receptor; VEGF, vascular endothelial growth factor.

The spatiotemporal heterogeneity of TASCs not only drives tumor progression but also presents a major obstacle to successful therapy. A key challenge is that TASCs dynamically remodel the TME during therapeutic interventions, thereby driving the emergence of therapeutic resistance (Table 4). For example, CAF-secreted hepatocyte growth factor (HGF) is responsible for resistance in *BRAF*-mutated melanoma [170] and thyroid anaplastic carcinoma [171]. Therefore, targeting the context-dependent functions of TASCs is crucial for overcoming therapeutic failure. To address this, Fig. 6 outlines promising therapeutic avenues that exploit this spatiotemporal heterogeneity. Furthermore, multiple clinical trials evaluating TASC-targeted therapeutic strategies are ongoing (Table 5).

**Table 4.** Therapy resistance caused by TASCs

Effect on cancer cells	TASCs	Cancer type	Upstream pathway	Molecule secreted by TASCs	Targeting therapy	Refs.
Antiangiogenic resistance	CAFs	Colorectal cancer	RAS signaling	NA	Antiangiogenic and RAS inhibitor	[274]
	CAFs	Hepatocellular carcinoma	SPP1/RAF/MAPK and PI3K/AKT/mTOR signaling	SPP1	Inhibiting SPP1	[275]
	CAPs	Thyroid carcinoma	TSP-1/TGF-β1/pERK1/pAKT/pSMAD3	TSP-1, TGF-β1	Inhibiting TSP-1/TGFβ1 axis	[276]
	PDGFR-β^+^ GPR91^+^ pericyte	Clear cell renal carcinoma	NA	Methionine	GRP91 antagonists	[277]
Chemoresistance	CAFs	Bladder cancer	miR-146a-5p/ARID1A/SOCS1/STAT3 signaling and miR-146a-5p/AMPKα2/mTOR signaling	miR-146a-5p	Inhibiting miR-146a-5p	[278]
	CAFs	Breast cancer	NA	C1ra, C1s2, C2, C3	Chemotherapy and complement receptor antagonist	[279]
	CAAs	Breast cancer	Doxorubicin efflux	The transport-associated major vault protein	Inhibiting MVP	[280]
	CAAs	Colorectal cancer	MTTP/PRAP1/E-box/glutathione peroxidase 4 and xCT	Exosomal MTTP	Targeting MTTP	[281]
	CD146^+^ pericyte	Glioblastoma	CCL5-CCR5-DDR signaling	CCL5	CCR5 antagonist MVC and temozolomide	[282]
	CA-MSCs	Acute myeloid leukemia	IL-6/JAK2/STAT3 signaling	IL-6	JAK2/STAT3 pathway inhibitor AG490	[283]
	myCAFs	Esophageal cancer	ECM remodeling	NA	Targeting S100A8–CD147 pathway	[284]
	TECs	Urothelial carcinoma	NA	ABCB1	ABCB1 inhibitor and paclitaxel	[285]
	TECs	Glioblastoma	Wnt/β-catenin signaling	NA	Wnt inhibition and temozolomide chemotherapy	[286]
	TSPAN8^+^ CAFs	Breast cancer	TSPAN8–SIRT6–MAPK11 signaling	SASP (IL-6, IL-8)	Anti-TSPAN8 antibody and SIRT6 activator MDL-800	[287]
Immune checkpoint blockade resistance	CAFs	Urothelial bladder cancer	NNMT/SAA	NA	Inhibitor 5-amino-1-methyl quinolinium iodide	[57]
	CAFs	Various tumor types	PI3K/AKT signaling	Insulin-like growth factor 2	Inhibiting the IGF2/IGF1R axis with the inhibitor linsitinib	[288]
	CXCL12^+^ TECs	Hepatocellular carcinoma	Inhibiting CD8^+^ cytotoxic T cells and attracting MDSCs	CXCL12	Bispecific antibody targeting CXCL12 and PD-1	[112]
	POSTN^+^ CAFs	Hepatocellular carcinoma	IL-6/STAT3 signaling	POSTN	Disturbing interactions between POSTN^+^ CAFs and SPP1^+^ macrophages	[88]
	THBS2^+^ mCAFs	Gastric cancer	C3–C3AR1 axis	C3a receptor 1	Blocking C3–C3AR1	[289]

ABCB1, ATP-binding cassette subfamily B member 1; AKT, protein kinase B; AMPKα2, AMP-activated protein kinase α2; ARID1A, AT-rich interaction domain 1A; C1ra, complement C1r subcomponent A; C1s2, complement C1s subcomponent 2; C2, complement C2; C3, complement C3; C3AR1, complement C3a receptor 1; CAAs, cancer-associated adipocytes; CAFs, cancer-associated fibroblasts; CA-MSCs, carcinoma-associated mesenchymal stem cells; CAPs, cancer-associated pericytes; CCL5, C–C motif chemokine ligand 5; CCR5, C–C motif chemokine receptor 5; CD146, cluster of differentiation 146; DDR, discoidin domain receptor; ECM, extracellular matrix; ERK1, extracellular signal-regulated kinase 1; GPR91, G protein-coupled receptor 91; IGF1R, insulin-like growth factor 1 receptor; IGF2, insulin-like growth factor 2; IL-6, interleukin-6; IL-8, interleukin-8; MAPK, mitogen-activated protein kinase; MAPK11, mitogen-activated protein kinase 11; MDSCs, myeloid-derived suppressor cells; MET, methionine; miR-146a-5p, microRNA-146a-5p; mTOR, mammalian target of rapamycin; MVP, major vault protein; myCAFs, myofibroblastic cancer-associated fibroblasts; NA, not analyzed; NNMT, nicotinamide N-methyltransferase; PD-1, programmed cell death protein 1; PDGFR-β, platelet-derived growth factor receptor β; PI3K, phosphoinositide 3-kinase; POSTN, periostin; pSMAD3, phosphorylated mothers against decapentaplegic homolog 3; RAF, rapidly accelerated fibrosarcoma kinase; RAS, rat sarcoma virus GTPase; SAA, serum amyloid A; SASP, senescence-associated secretory phenotype; SIRT6, sirtuin 6; SMAD3, mothers against decapentaplegic homolog 3; SOCS1, suppressor of cytokine signaling 1; SPP1, secreted phosphoprotein 1; STAT3, signal transducer and activator of transcription 3; TASCs, tumor-associated stromal cells; TECs, tumor-associated endothelial cells; TGF-β1, transforming growth factor-β1; THBS2, thrombospondin 2; TSP-1, thrombospondin-1; TSPAN8, tetraspanin 8; Wnt, wingless-related integration site; xCT, cystine/glutamate antiporter

**Table 5.** Clinical trials targeting TASCs

Target	Drug	Combination therapy	Conditions	Phase	Status	**Trial number**
*CAF-related clinical trials*						
B7-H3	CD276 CAR-T	No	Advanced pancreatic carcinoma	I/II	Unknown	NCT05143151
FAP	AVA6000	No	Salivary gland tumor, urothelial carcinoma, ovarian carcinoma, breast cancer, soft tissue sarcoma	I	Recruiting	NCT04969835
	Nectin4/FAP-targeted fourth-generation CAR-T cells	No	Nectin4-positive advanced malignant solid tumor	I	Unknown	NCT03932565
	OMTX705	Pembrolizumab	Advanced solid tumor	I	Recruiting	NCT05547321
	Simlukafusp alfa (RO6874281)	Atezolizumab	Advanced/metastatic head, neck, esophageal and cervical cancers	II	Completed	NCT03386721
	68Ga-FAPI	No	Malignant neoplasm	Not applicable	Recruiting	NCT05034146
FGFR	AZD4547	Docetaxel	Recurrent non-small cell lung cancer, squamous cell lung cancer	I/II	Completed	NCT01824901
	HMPL-453	Gemcitabine and cisplatin, toripalimab, docetaxel	Solid tumor	I/II	Recruiting	NCT05173142
	NJ-42756493 (erdafitinib)	No	Neoplasm	II	Completed	NCT02699606
	Rogaratinib (BAY1163877)	Chemotherapy	Carcinoma	II/III	Completed	NCT03410693
	IMGN388	No	Solid tumor	I	Completed	NCT00721669
Integrin αVβ3	ProAgio	No	Advanced pancreatic cancer, solid tumor	I	Recruiting	NCT05085548
	177Lu-AB-3PRGD2	No	Integrin αVβ3- positive tumors	I	Recruiting	NCT05013086
LOX	PXS-5505	No	Myelofibrosis	I/II	Active, not recruiting	NCT04676529
	Simtuzumab	Gemcitabine	Pancreatic cancer	II	Completed	NCT01472198
	Bintrafusp alfa	Definitive chemoradiation	Carcinoma, squamous cell, esophageal cancer	Not applicable	Recruiting	NCT04481256
		No	Uterine cervical neoplasms	II	Completed	NCT04246489
PD-L1/TGF-β	HCB301	No	Advanced solid tumor	I	Not yet recruiting	NCT06487624
	Y101D	No	Metastatic or locally advanced solid tumors	I	Active, not recruiting	NCT05028556
Reprogramming CAFs	All-trans retinoic acid	Gemcitabine, nab-paclitaxel	Pancreatic cancer	I	Completed	NCT03307148
			Pancreatic cancer	II	Active, not recruiting	NCT04241276
		Nivolumab	Pancreatic cancer	I	Not completed	NCT05482451
TGF-β	Fresolimumab	Radiation therapy	Metastatic breast cancer	II	Completed	NCT01401062
	Galunisertib	Paclitaxel	Estrogen receptor negative, HER2/neu negative, progesterone receptor negative recurrent breast carcinoma	I	Completed	NCT02672475
	Vactosertib	Pomalidomide	Multiple myeloma	I	Completed	NCT03143985
		Imatinib	Desmoid tumor	I/II	Completed	NCT03802084
Vitamin D receptor	Paricalcitol	Gemcitabine, nab-paclitaxel	Pancreatic cancer	I	Terminated	NCT03520790
		Pembrolizumab	Pancreatic cancer, pancreatic adenocarcinoma	II	Completed	NCT03331562
*TEC-related clinical trials*						
Ang	AMG780	No	Advanced solid tumors	I	Terminated	NCT01137552
		Bevacizumab	Metastatic colorectal cancer	II	Unknown	NCT01249521
	Zansecimab (Y3127804)	Ramucirumab, paclitaxel	Solid tumors	I	Completed	NCT02597036
Ang/Tie	Trebananib (AMG 386)	No	Neoplasms, advanced solid	I	Completed	NCT02525536
		Pazopanib hydrochloride, sorafenib tosylate	Advanced renal cell carcinoma, advanced sarcomatoid renal cell carcinoma	II	Completed	NCT01664182
		Pembrolizumab	Advanced solid tumor	I	Active, not recruiting	NCT03239145
CAR-T therapy	Claudin18.2 CAR-T	No	Advanced pancreatic carcinoma, advanced gastric carcinoma	Not applicable	Recruiting	NCT05620732
	Dual-targeting CLDN18.2 and PD-L1 CAR-T cells	No	Advanced solid tumor	I	Recruiting	NCT06084286
	Anti-VEGFR2 CAR-T cells CD8 plus PBL	Cyclophosphamide, aldesleukin, fludarabine	Metastatic cancer, metastatic melanoma, renal cancer	I/II	Terminated	NCT01218867
PD-1/VEGFA/CTLA-4	CS2009	No	Advanced solid tumors	I	Recruiting	NCT06741644
	Lenvatinib	Camrelizumab	Hepatocellular carcinoma	I/II	Unknown	NCT04443309
		Toripalimab	Advanced biliary tract cancer	II	Unknown	NCT04211168
VEGF/PD-1	Ivonescimab (AK112)	Chemotherapy	Pretreated pleural mesothelioma	II	Recruiting	NCT06875076
	JS-207	Pemetrexed, carboplatin, or cisplatin	Nonsquamous non-small cell lung cancer	II	Not yet recruiting	NCT06868836
		No	Advanced malignant tumor	I	Recruiting	NCT06022250
	RC-148	Carboplatin and paclitaxel/pemetrexed	Non-small cell lung cancer	I	Not yet recruiting	NCT06883630
	SCTB-14	No	Advanced malignant solid tumors	I/II	Not yet recruiting	NCT06304818
	SSGJ-707	Bevacizumab, oxaliplatin	Metastatic colorectal cancer	II	Recruiting	NCT06493760
		Carboplatin, pemetrexed	First-line advanced non-small cell lung cancer patients	II	Recruiting	NCT06412471
VEGF/PD-L1	Bevacizumab	Atezolizumab, proton, radiotherapy	Hepatocellular carcinoma nonresectable	II	Recruiting	NCT06133062
		No	Metastatic alveolar soft part sarcoma, unresectable alveolar soft part sarcoma	II	Active, not recruiting	NCT03141684
		No	Hepatocellular carcinoma, non-small cell lung cancer metastatic, liver metastases	II	Recruiting	NCT04563338
	B1926	No	Advanced colorectal cancer	II	Not yet recruiting	NCT06838546
	HB0025	Pemetrexed, paclitaxel, carboplatin	Advanced non-small cell lung cancer, advanced endometrial cancer	I/II	Recruiting	NCT06758557
	IMM2510	Chemotherapy	Non-small cell lung cancer, triple-negative breast cancer	II	Not yet recruiting	NCT06746870
	Ivonescimab (AK112)	Placebo	Nonsquamous non-small cell lung cancer	III	Active, not recruiting	NCT06396065
	PM8002/BNT327	Paclitaxel, topotecan	Small cell lung cancer	III	Recruiting	NCT06616532
		Etoposide	Extensive-stage small cell lung cancer, small cell lung cancer	II	Active, not recruiting	NCT06449209
		Pm1009, atezolizumab	Hepatic cell carcinoma, liver cancer	I/II	Not yet recruiting	NCT06584071
	Ramucirumab (LY3009806)	Medi4736	Gastric cancer, gastroesophageal junction adenocarcinoma, non-small cell lung cancer, hepatocellular carcinoma	I	Completed	NCT02572687
	Zanzalintinib (XL-092)	Durvalumab, tremelimumab	Hepatocellular carcinoma	II	Recruiting	NCT06698250
*CAP-related clinical trials*						
PDFGR	Anlotinib	Cadonilimab	Esophageal carcinoma	II	Not yet recruiting	NCT06681285
		Eribulin	Breast cancer	II	Not yet recruiting	NCT06678230
	ETN101	No	Hepatocellular carcinoma, advanced hepatocellular carcinoma, liver cancer	I	Recruiting	NCT06326502
	Famitinib	Adebrelimab	Thyroid cancer	II	Not yet recruiting	NCT06146985
	Nintedanib	No	Castleman disease	II	Not yet recruiting	NCT06643091
	Vorolanib	No	Advanced non-small cell lung cancer, recurrent or metastatic lung cancer	II	Recruiting	NCT06728852
		Sintilimab	Renal cell carcinoma	II	Not yet recruiting	NCT06523049
*CA-MSC-related clinical trials*						
Engineered MSCs	BM-MSC-INFβ	No	Ovarian cancer	I	Completed	NCT02530047
	MSC-DNX-2401	No	Recurrent anaplastic astrocytoma, recurrent glioblastoma, recurrent gliosarcoma, recurrent malignant glioma	I	Recruiting	NCT03896568
	MSC-IFN-α	Nabpaclitaxel, cyclophosphamide and anti-PD-1 monoclonal antibody	Locally advanced or metastatic solid tumors	I/II	Recruiting	NCT05699811
	MSC-L	No	Advanced colorectal cancer	I	Recruiting	NCT06446050
	MV-NIS Infected MSC	No	Fallopian tube clear cell adenocarcinoma	I/II	Active, not recruiting	NCT02068794
Exosomes	Exosomes with KRAS G12D siRNA	No	Metastatic pancreatic adenocarcinoma pancreatic ductal adenocarcinoma	I	Active, not recruiting	NCT03608631
MSCs	Allogeneic human MSCs	No	Prostate cancer	I	Terminated	NCT01983709
	Cord blood MSCs	Neurorege scaffold	Rectal cancer	I/II	Recruiting	NCT02648386
	Cord blood-derived MSCs	No	Hematopoietic and lymphoid cell neoplasm, malignant solid neoplasm	I/II	Recruiting	NCT04565665

AK112, ivonescimab; Ang, angiopoietin; CAFs, cancer-associated fibroblasts; CA-MSCs, carcinoma-associated mesenchymal stem cells; CAPs, cancer-associated pericytes; CAR-T, chimeric antigen receptor T cell; CTLA-4, cytotoxic T lymphocyte-associated protein 4; DNX-2401, delta-24-rgd oncolytic adenovirus; ECM, extracellular matrix; FAP, fibroblast activation protein; FAPI, fibroblast activation protein inhibitor; FGFR, fibroblast growth factor receptor; Ga, gallium; HER2, human epidermal growth factor receptor 2; IFN, interferon; IL, interleukin; KRAS, Kirsten rat sarcoma viral oncogene homolog; LOX, lysyl oxidase; MSC, mesenchymal stromal cell; MV-NIS, measles virus encoding sodium iodide symporter; PD-1, programmed cell death protein 1; PD-L1, programmed cell death ligand 1; PDGFR, platelet-derived growth factor receptor; RGD, arginine–glycine–aspartic acid; siRNA, small interfering RNA; TGF-β, transforming growth factor-β; TECs, tumor-associated endothelial cells; VEGF, vascular endothelial growth factor; VEGFR2, vascular endothelial growth factor receptor 2

### Target TASCs in the context of temporal heterogeneity

#### Targeting TASCs sculpts antitumor niches

Employing molecular markers of TASCs to guide immunotherapy is pivotal for tumor treatment. Accordingly, a number of clinical trials have been designed to target these specific molecular markers, as summarized in Table 5. However, the functional heterogeneity of TASCs necessitates caution, as monolithic inhibition or depletion strategies may compromise therapeutic efficacy and potentially promote tumor progression. Here, we use CAFs as a prominent example to address this challenge. In genetically engineered mouse models of PDAC, eliminating FAP^+^ CAFs resulted in improved survival, whereas removing α-SMA^+^ CAFs led to reduced survival [172]. This complexity was further illustrated by the expression of yes-associated protein 1 (YAP1), a key regulator in CAF phenotypic transformation. YAP1 activation drove the differentiation of extracellular matrix-associated CAFs (ECM-CAFs), whereas YAP1 silencing conversely reprogrammed CAFs toward a lymphatic-like phenotype. In *Yap1^flox/flox^* mice, selective YAP1 knockdown in ECM-CAFs resulted in damaged ECM immune barriers and stabilized lymphatic vessels. This synergistic effect boosted CD8^+^ T cell trafficking, thereby augmenting the efficacy of anti-PD-1 therapy [173]. Unfortunately, manipulating the phenotypic transformation of CAFs does not always yield beneficial outcomes. Blocking the Hedgehog pathway inhibited myCAFs but increased the proportion of iCAFs, resulting in a stronger immunosuppressive microenvironment [174].

The intricate and even contradictory outcomes of pathway-specific interventions have motivated the exploration of broader regulatory strategies, such as epigenetic reprogramming. DNA methylation and histone deacetylation constitute pivotal regulatory mechanisms within TASCs. Clinically available agents include approved DNA methyltransferase (DNMT) inhibitors (e.g., 5-azacitidine, 5-aza-2′-deoxycytidine, and GSK3685032) and histone deacetylase (HDAC) inhibitors (e.g., vorinostat, panobinostat, and givinostat) [175]. Nevertheless, the efficacy of these agents in remodeling TASCs remains inadequately characterized.

Recently, mechanistic insights for novel epigenetic therapeutic strategies have been gleaned, typically in CAFs and TECs. CAFs display heterogeneous DNA methylation patterns, but harbor evolutionarily conserved hypomethylated CpG sites. These sites represent actionable therapeutic targets [176]. The ectopic expression of DNMT1 has been identified [177]. Pharmacological inhibition using 5-aza-2′-deoxycytidine attenuated CAF malignancy and showed therapeutic benefits in PDAC murine models [178]. Notably, epigenetic alterations within CAFs are also involved in angiogenesis [179], metabolic reprogramming [180], and tumor invasion [181]. Despite their therapeutic potential, rigorous preclinical validation and clinical trial data are currently limited.

TECs exhibit distinct DNA methylomes, with promoter hypomethylation linked to pro-angiogenic activation and tumor invasion [182]. Furthermore, HDAC-dependent histone deacetylation has been shown to silence adhesion molecules (e.g., ICAM1 and VCAM1) [183], rationalizing combinatorial AAT and epigenetic targeting. Ongoing related combined strategies include 5-azacitidine and anti-VEGF monoclonal antibody (bevacizumab) for renal cell carcinoma (NCT00934440) and vorinostat and epidermal growth factor receptor (EGFR) inhibitor (gefitinib) for NSCLC (NCT02151721). In summary, targeting TASCs through their molecular markers and epigenetic machinery has profound translational potential. Combining epigenetic therapies with existing modalities such as immunotherapy and anti-angiogenic treatment represents a promising frontier for improving cancer treatment outcomes.

#### Turning enemies into friends: Reprogramming TASCs

Beyond eradicating TASCs, reprogramming them from protumorigenic to a quiescent or antitumorigenic state represents an advanced therapeutic strategy. This approach aims to normalize the TME, thereby attenuating stromal support for malignancy and enhancing the efficacy of concomitant therapies.

##### Reprogramming CAFs

Reprogramming CAFs as an adjuvant strategy has moved from the bench to the bedside. In PDAC, vitamin A deficiency and vitamin D receptor (VDR) expression are key triggers for the activation of pancreatic stellate cells (PSCs). Preclinical experiments have confirmed that normalizing murine PSCs with the external VDR ligand calcipotriol markedly improved survival by 57% when combined with chemotherapy [184,185]. Clinically established all-trans retinoic acid and lenvatinib bind to the retinoic acid receptor β (RAR-β) and FGFR, respectively. These interactions regulate CAFs and hamper tumor progression [186]. Novel therapeutic approaches in preclinical studies have emerged. An NADPH (reduced form of nicotinamide adenine dinucleotide phosphate) oxidase 4 (NOX4) inhibitor was shown to normalize CAFs in vivo, improving CD8^+^ T cell infiltration [164]. To precisely reprogram CAFs, EVs modified with integrin α5-targeting peptides have been developed. These EVs deliver cargo such as miR-138-5p and pirfenidone to inhibit CAF activation by blocking the TGF-β signaling pathway [187].

##### Reprogramming TECs

Approaches to reprogramming TECs mostly focus on AAT, which are described in Targeting the tumor vasculature starves tumors. Beyond AAT, an emerging strategy involves reprogramming TECs into HEVs. Evidence indicates that during cancer immunotherapy, infiltrating CD8^+^ T cells and NK cells activate LTβR, which facilitates post-capillary venule transformation into HEVs. HEVs, in turn, attract T cells to hamper tumor progression [17]. Therefore, actively reprogramming TECs into HEVs represents a promising future therapeutic direction.

##### Reprogramming CAAs

Although the basic research on CAAs is still weak, breakthroughs in translational research have laid the key technological foundations for CAA reprogramming strategies. A study developed engineered adipocytes with clustered regularly interspaced short palindromic repeat activation (CRISPRa) that exhibited high metabolism, dramatically inhibiting the proliferation of cancer cells. This antitumor effect was mediated by the restriction of glucose and lipid metabolism in tumor cells, as demonstrated in murine models of PDAC and BC [188].

##### Reprogramming CA-MSCs

Accumulating evidence demonstrates that the carcinogenic effects of CA-MSCs can be rewired by exogenous signals. In mouse melanoma, studies have shown that CA-MSCs recruit macrophages and MDSCs under the orchestration of NF-κB signaling. Retinoic acid blocks the NF-κB pathway, thereby preventing immune cells and reversing tumor progression [189]. Treatment with IL-12 in TNBC mice counteracted the tumor-promoting effects of CA-MSCs by up-regulating IL-12 and IFN-γ expression [190]. What is more, the inhibition of activated signaling is beneficial for tumor immunotherapy. YAP signaling was activated during the CA-MSC phenotypic transition in patients with lymph node metastasis from GC. Verteporfin successfully inhibited YAP signaling and reprogrammed CA-MSCs in mice, disrupting tumor metastasis and invasion [191].

The reprogramming of diverse TASCs has matured from conceptual ideas to tangible strategies. These approaches seek to normalize the entire tumor ecosystem, offering a powerful means to overcome therapeutic resistance.

#### Targeting the tumor vasculature starves tumors

Pathological blood vessels, as key drivers of cancer progression, are primarily addressed through anti-angiogenesis and tumor vascular normalization strategies. Anti-angiogenic drugs restrain tumor angiogenesis by blocking critical signaling pathways. Targeted therapies against VEGF and PDGF are well established, with agents such as bevacizumab, ranibizumab, and olaratumab already on the market [192]. However, resistance to these AATs has emerged as a major clinical challenge, limiting their long-term efficacy. To address the challenge, targeting CAPs may yield beneficial outcomes. Paracrine signaling from CAPs supports tumor cell proliferation, survival, and migration. Studies in multicancer mouse models have discovered that CAPs stimulate tumor cell proliferation through paracrine secretion of high-mobility group protein 1 (HMGB1) [193]. The limited efficacy of imatinib and sunitinib in treating tumors may arise from this mechanism [194,195]. Therefore, targeting CAPs for anti-angiogenic activity requires further refinement to minimize adverse outcomes.

In response to the limitations of AAT, tumor vascular normalization has evolved into a complementary strategy. Instead of destroying vessels, this approach aims to improve vascular permeability, increase pericyte coverage, and promote vascular maturation [196]. Directly modifying TECs is a feasible strategy. In tumor-bearing mice, salvianic acid A promoted tight junctions between TECs, reversing aberrant structures and functions of tumor vessels through the pyruvate kinase muscle isozyme 2 (PKM2)/β-catenin/claudin-5 signaling axis [197]. Collectively, these multifaceted strategies targeting tumor vasculature are pivotal for overcoming resistance and improving cancer treatment outcomes.

#### TASC metabolic reprogramming halts tumor progression

Targeting metabolic pathways within TASCs holds profound therapeutic potential for improving tumor treatment and immune responses, but relevant studies remain preclinical.

##### Targeting CAF-associated metabolic pathways

Evidence from in vivo research has indicated that interrupting the normal metabolism of CAF affects tumor outcomes. In PDAC, owing to the reduction in lactate from glycolysis, nerve invasion is prevented [198]. Consistent with this, pharmacological inhibition of the Src SH3 domain of the facilitative glucose transporter (GLUT1) has been shown to suppress glycolysis and deactivate CAFs [199]. In addition to glucose metabolism, lipid biosynthesis in CAFs represents a key metabolic vulnerability. CAFs up-regulate ATP citrate lyase (ACLY) to produce fatty acids for energy. Therefore, the combination of ACLY inhibitor NDI-091143 and antitumor drugs (e.g., Adriamycin or paclitaxel) has been demonstrated to disrupt normal metabolism [200].

##### Targeting TEC-associated metabolic pathways

Inhibiting specific metabolic enzymes in TECs could enhance the efficacy of immunotherapy by improving tumor vascular function. For instance, osimertinib represses glyceraldehyde-3-phosphate dehydrogenase (GAPDH), thereby reducing lactate secretion from TECs and reversing the acidic TME [201]. Phosphoglycerate dehydrogenase (PHGDH) is crucial for nucleotide synthesis, serine metabolism, and glycolysis. Inhibitors such as WQ2201 normalize tumor vessels and enhance T cell infiltration, supporting chimeric antigen receptor T cell (CAR-T) therapies in glioblastoma [202].

##### Targeting CAA-related metabolic pathways

Distinct from traditional inhibition approaches, enhancing metabolism in CAAs seems to be effective. The previously mentioned engineered CAAs with high metabolism are robust evidence. Moreover, a fructose diet induced adipocytes to unleash leptin via a mechanistic target of rapamycin complex 1 (mTORC1)-dependent pathway. High levels of leptin boosted the antitumor immune response of CD8^+^ T cells [203].

Despite unclear clinical applications, these findings highlight the therapeutic potential of targeting TASC metabolic pathways, which reshape the TME and enhance immunotherapy. Bridging compelling preclinical insights into clinical translation represents a next step in cancer treatment.

#### Targeting ECM curbs spread and supports immunity

Restraining ECM deposition signals and fostering degradation are crucial strategies for ameliorating cancer immunotherapy. In the context of ECM deposition, LOX is a crucial factor that promotes matrix rigidity. Preclinical studies have shown that specific inhibitors targeting LOX play a role in overcoming immunotherapy resistance [204,205]. β-Aminopropionitrile (BAPN), the first LOX inhibitor, is widely used in animal experiments to prohibit collagen cross-linking. However, its high toxicity has precluded clinical application [206]. PXS-5505 is a potent pan-LOX inhibitor that perturbs cancer cell invasion and enhances the effect of chemotherapy. The therapeutic feasibility of PXS-5505 in combination with gemcitabine in pancreatic cancer has been approved [207]. However, there are currently no effective blockers on the market.

Degrading key ECM components represents a promising strategy to inhibit tumor progression and improve drug delivery. This can be achieved by leveraging bioactive enzymes, such as hyaluronidases, collagenases, and MMPs [208]. A notable advancement in this area involves engineering CAR-T cells with Notch receptors, enabling them to eradicate the ECM by inducing the secretion of heparinase and MMPs [209]. Dual strategies focusing on ECM deposition and ECM degradation hold profound promise for normalizing the tumor stroma and augmenting the response to immunotherapy.

### Spatial heterogeneity and clinical relevance

Advances in spatial encoding have accelerated the exploration of the internal architecture of tumors, and the exploration of spatial heterogeneity has deepened the understanding of cellular ecological niches, which are instructive for diagnosis and treatment.

#### Tumor niches unlock patient prognostic stratification

Recent advances in single-cell resolution and spatial localization technologies have unveiled novel dimensions for prognostic evaluations within the TME. These approaches allow for sophisticated analyses, from the spatial distribution of individual cell subtypes to the architecture of the TME, providing critical insights for patient stratification and clinical decision-making. Here, we synthesize key findings across 3 main areas: the prognostic value of specific cell subtypes, the utility of spatial biomarkers, and the comprehensive landscape of the TME.

The presence and localization of specific stromal cell subpopulations enable the prediction of prognoses. In the context of CAFs, an analysis of 1,070 NSCLC patients has delineated distinct prognostic values for different CAF subpopulations based on their spatial localizations. Those at the tumor–stroma interface, consisting of iCAFs, interferon-responsive CAFs (ifnCAFs) (a subset of CAFs highly responsive to interferon signaling), and α-SMA^+^ CAFs, were associated with longer patient survival. In contrast, prometastatic subpopulations within the tumor core or deep stroma, comprising tumor-like CAFs (tCAFs) (a type of CAF expressing tumor stem cell-related markers), hypoxic tCAFs, and matrix CAFs, were linked to poor prognosis [210]. The balance of TEC subpopulations also influences clinical outcomes. Pan-cancer studies have identified 2 key subpopulations: pro-angiogenic E02-tip-CXCR4 and immune-favorable E06-vein-selectin E (SELE). An increased ratio of E02 to E06 was positively correlated with improved survival rates. Owing to its role in leukocyte adhesion, the E06 subpopulation also predicted immune checkpoint inhibitor (ICI) response [106]. More importantly, in clinical practice, Jiang et al. [211] established the TEC score based on differentiation genes, which has been validated as an independent poor prognostic factor for intrahepatic cholangiocarcinoma (ICC).

Beyond single cells, spatial structures within tumor tissues serve as powerful spatial biomarkers and facilitate patient stratification. TLSs have emerged as unique spatial biomarkers due to their prominent role in predicting antitumor immune responses. The densities, cellular compositions, and spatial distributions of TLSs correlate strongly with the sensitivity to PD-1/PD-L1 blockade [212,213]. Clinical evidence from a trial (NCT02534649) has confirmed that TLS-positive patients exhibited prolonged median overall survival (24.8 versus 13.3 months) and progression-free survival (6.1 versus 2.1 months) following anti-PD-1/L1 therapy. Moreover, the spatial location of TLSs is crucial, as it represents distinct functional states. In patients with ICC, the presence of intratumoral TLSs extends overall survival, whereas the presence of peritumoral TLSs shortens it. Immunoclassification based on TLS spatial distributions has outperformed the traditional tumor node metastasis classification (TNM) classification in predicting overall survival [214]. The CAF-Epi-Immune score in ESCC further exemplifies the concept of composite spatial scoring systems. This score is biologically grounded in the observation that spatial niches formed by CAFs and epithelial cells drive tumor invasion through ECM remodeling and immunosuppression [215,216].

Finally, a comprehensive assessment of the TME further optimized the hierarchical model. The stromal score and immune score, which reflect the infiltration of stromal and immune cells, respectively, are regarded as valuable tools for survival predictions. High stromal scores are associated with poor prognoses and mediated resistance to immunotherapy; immune scores show the opposite trend [217–219]. Additionally, the combination of these scores allows for more refined patient stratification [220]. Recent pan-cancer analyses have advanced this concept by identifying conserved TME subtypes. For instance, patients with immune-enriched, fibrotic types showed the best response to ICIs; those with immune-enriched, nonfibrotic types had the longest survival; and those with fibrotic and desert types exhibited ICI resistance and poor prognosis, respectively. Dynamic monitoring of these subtypes can inform treatment decisions and precision personalized medicine [221]. Another compelling example from cervical squamous cell carcinoma has illustrated how spatial atlases reveal therapeutic vulnerabilities. The MP6 subtype (epithelial–keratinocyte) drove immune suppression via CAF activation, whereas the MP7 subtype (epithelial–immune) promoted protective immune cell infiltration. Targeting a key driver (fatty acid-binding protein 5) in the MP6 subtype facilitated the transition between tumor states, highlighting a novel therapeutic avenue [222]. These dynamic models are progressively moving from descriptive snapshots toward clinical tools for precision medicine.

As spatial technologies become more accessible and standardized, their integration into clinical trials and routine diagnostics will be crucial for realizing personalized cancer therapy. Future efforts should focus on validating spatial biomarkers prospectively and developing targeted interventions. Ultimately, the gap between spatial biology and clinical practice should be bridged to improve patient outcomes.

#### Cellular interactions within the TIF reveal new therapeutic targets

Therapeutic strategies targeting FAP^+^ CAFs have attracted increasing attention due to their central role in shaping the TIF and promoting immunosuppression [100,223]. A range of modalities is currently under investigation, from immunotherapies to signaling regulation. Multiple FAP-directed strategies have been developed, ranging from biologics such as monoclonal antibodies and DNA vaccines to advanced cell therapies such as CAR-T cells. Typically, engineering CAR-T cells to secrete anti-FAP molecules has successfully eradicated the PDAC stroma in preclinical models [224]. Beyond direct targeting, modulating the signaling activity of FAP^+^ CAFs also shows promise. Blocking CXCL12 signaling from these cells has been demonstrated to synergize with anti-PD-L1 therapy and improve treatment outcomes [225]. In contrast, activating specific signaling combined with anti-FAP has potent antitumor efficacy. FAP-IL2v, comprising an interleukin-2 variant (IL-2v) fused to an antibody targeting FAP, triggers JAK-STAT signaling and activates CD8^+^ T cells [226]. The representative drug, simlukafusp slfa, is currently undergoing phase I/II clinical trials and is poised to offer a transformative strategy for combating advanced solid tumors [227–229]. To conclude, the spatial delineation of FAP^+^ CAF niches and their molecular interactions provides a roadmap for developing spatially targeted strategies.

### Therapies targeting TASCs in combination with immunotherapies

Owing to their pivotal role in immune regulation, targeting TASCs provides a powerful adjunct to cancer immunotherapy. Here, we introduce several synergistic strategies that increase immunotherapeutic efficiency, including specific CAF targeting, AAT, and ECM remodeling. According to clinical trial datasets for several solid tumors, CAFs with leucine-rich repeat-containing 15 (LRRC15) signatures drive resistance to anti-PD-L1 therapy. In response, the antitumor activity of the specific antibody drug ABBV-085 has been evaluated in several clinical trials (e.g., NCT02565758 and NCT02565758) [230].

AAT reprograms the immunosuppressive TME to enhance the efficacy of ICIs. Therapy combining AAT with ICI has demonstrated new synergistic potential in clinical trials. The Impower150 study (i.e., NCT02366143) evaluated the clinical efficacy of the combination of atezolizumab (an anti-PD-L1 antibody) and bevacizumab (a VEGF inhibitor) in NSCLC. This combination therapy remarkably improved both progression-free survival and overall survival in patients. Based on these positive findings, the U.S. Food and Drug Administration (FDA) has approved this regimen as a first-line treatment option [231]. Another combination of carrilizumab (an anti-PD-L1 antibody) and apatinib (a VEGFR inhibitor) has exhibited therapeutic potential for several tumor types, covering NSCLC, HCC, and oral squamous cell carcinoma (OSCC), in preclinical models and clinical trials (e.g., NCT04379739, NCT04297202, and NCT04393506) [232–234].

Furthermore, accelerating ECM degradation disrupts the immune barrier in the TME. In murine models of BC, the nanomedicine dasatinib reduced ECM matrix secretion and improved matrix permeability, providing a novel method for PD-1/L1 combination therapy [235]. Overall, integrating TASC-targeting approaches into current immunotherapeutic regimens is poised to redefine treatment standards and improve survival outcomes for a broader population of cancer patients.

## Conclusion and Perspectives

The intricate spatiotemporal heterogeneity of TASCs within the TME has been investigated in this review. We have provided insights into the evolution and functional characteristics of TASCs, with a focus on their spatial distribution and immunosuppression.

### Key findings and challenges

TASCs are the main components of the TME and exhibit spatiotemporal heterogeneity. The dynamic evolution and spatial organization of TASCs are fundamental drivers of tumor initiation, progression, and metastasis. Through intricate intercellular crosstalk, TASCs construct diverse functional niches that profoundly shape the antitumor immune response. Consequently, dissecting the heterogeneity of TASCs has emerged as a pivotal breakthrough for advancing cancer immunotherapy.

Nevertheless, many outstanding challenges still exist. First, the lack of a unified classification system for TASC subpopulations poses great difficulties for research and clinical translation. Further clarification of the molecular markers, functions, and origins of TASCs across diverse tumor types and therapeutic scenarios is essential [17]. Employing the classic approach that combines marker genes with functional annotation may improve the accuracy and completeness of the annotations. Second, the scarcity of specific marker genes within stromal cells leads to ambiguous boundaries in delineating distinct cell populations. In particular, the gene expression profiles overlap in cell types, such as vCAFs and CAPs, myCAFs, and smooth muscle cells. This challenge not only complicates subpopulation clustering in research studies but also hampers the precision of targeted stromal cell therapies. In future targeted therapeutic studies, it is important to consider functional diversity. Finally, phenotypic shifts in TASCs may occur during treatment, contributing to resistance. Therefore, accelerating stromal cell heterogeneity research, especially in the translation of animal models to preclinical experiments, will be key. The resolution of the following outstanding questions will help decipher the heterogeneity of TASCs: How can the various classification criteria be harmonized? How can TASCs be precisely targeted in clinical applications without damaging stromal cells in normal tissues? How does the heterogeneity of TASCs change dynamically during tumor therapy?

### Technological advances and future directions

With the continuous development of scRNA-seq and ST technologies, the spatial architecture within tumors has gradually been resolved more precisely. These advances have led to revolutionary breakthroughs in the precise stratification of tumor patients, tumor grading, and treatment predictions. For example, Sorger and colleagues [236] have provided insights into tumor progression mechanisms and potential treatment strategies by mapping large-scale spatial profiles of CRC. However, effectively translating spatial information into clinical applications still faces many challenges, such as integrating data from different platforms and eliminating data bias. To address these issues, the combination of machine learning and spatial genomics has shown potential. By integrating multiple data types, the Tumoroscope platform identified colocalization and exclusion phenomena within tumor tissues [237]. Meanwhile, single-cell 3D imaging technology (mLSR-3D) combined with spatial data analysis revealed the spatial phenotypes of tumors at the cellular level, promoting the construction of tumor maps and accurate diagnosis [238]. To realize this potential, the systematic acquisition of high-quality spatial data across a wide spectrum of cancers is essential. A substantial number of clinical trials remain imperative to amass adequate spatial datasets. Multicenter validation and machine learning-driven identification of robust spatial signatures collectively enhance the clinical reliability of spatial information.

In the future, deciphering the spatial atlas of the TME is a promising direction. Continued progress in spatial profiling will critically illuminate the temporal progression of tumors and the spatiotemporal heterogeneity of TASCs. These advancements will provide new opportunities for clinical treatment, expanding the repertoire of precision medicine and targeted therapy.
